# Using the New “Life’s Essential 8” Metrics to Evaluate Trends in Cardiovascular Health Among US Adults From 2005 to 2018: Analysis of Serial Cross-sectional Studies

**DOI:** 10.2196/45521

**Published:** 2023-05-08

**Authors:** Cheng Li, Yanzhi Li, Min Zhao, Cheng Zhang, Pascal Bovet, Bo Xi

**Affiliations:** 1 Department of Epidemiology, School of Public Health, Qilu Hospital Cheeloo College of Medicine Shandong University Jinan China; 2 Department of Medical Statistics and Epidemiology, School of Public Health Sun Yat-sen University Guangzhou China; 3 Department of Nutrition and Food Hygiene, School of Public Health, Cheeloo College of Medicine Shandong University Jinan China; 4 Key Laboratory of Cardiovascular Remodeling and Function Research, Chinese Ministry of Education, Chinese National Health Commission and Chinese Academy of Medical Sciences, The State and Shandong Province Joint Key Laboratory of Translational Cardiovascu Shandong University Jinan China; 5 Center for Primary Care and Public Health (Unisanté) University of Lausanne Lausanne Switzerland

**Keywords:** trends, cardiovascular health, primordial prevention, adult, nutrition examination, survey, diet, physical activity, data collection, cross-sectional

## Abstract

**Background:**

The recently published “Life’s Essential 8” (LE8) by the American Heart Association has overcome some limitations in evaluating cardiovascular health (CVH) in the previous “Life’s Simple 7.”

**Objective:**

We aimed to examine the secular trends in CVH, as assessed by the LE8, in US adults from 2005 to 2018.

**Methods:**

Using cross-sectional data from the National Health and Nutrition Examination Survey between 2005-2006 and 2017-2018, we calculated the age-standardized mean scores of overall CVH and each of the LE8 components, where a higher score (range 0-100 points) means a better health status. A total of 21,667 adults aged 20-79 years were included in this analysis.

**Results:**

The overall CVH did not significantly change between 2005-2006 and 2017-2018 (65.5, 95% CI 63.9-67.1 to 65.0, 95% CI 62.8-67.1; *P*=.82). The individual metrics did not significantly change for diet (41.0, 95% CI 38.0-43.9 to 41.5, 95% CI 36.5-46.6; *P*=.94), physical activity (57.5, 95% CI 53.0-61.9 to 53.0, 95% CI 48.7-57.3; *P*=.26), and blood pressure (68.4, 95% CI 65.2-71.5 to 68.6, 95% CI 65.3-71.9, *P*=.35), improved for nicotine exposure (64.7, 95% CI 61.1-68.4 to 71.9, 95% CI 67.7-76.2; *P*<.001), sleep health (83.7, 95% CI 81.6-85.7 to 84.1, 95% CI 81.2-87.1; *P*=.006), and blood lipids (61.6, 95% CI 59.1-64.0 to 67.0, 95% CI 63.5-70.4; *P*<.001), and worsened for BMI (63.4, 95% CI 59.7-67.1 to 56.2, 95% CI 52.5-59.9; *P*<.001) and blood glucose (83.9, 95% CI 82.4-85.4 to 77.4, 95% CI 74.5-80.3; *P*<.001).

**Conclusions:**

According to the LE8, the overall CVH did not change among US adults from 2005 to 2018, as well as 3 components (diet, physical activity, and blood pressure). Other metrics such as nicotine exposure, blood lipids, and sleep health improved, while BMI and blood glucose deteriorated over time.

## Introduction

Cardiovascular disease (CVD) is a major public health issue worldwide [1]. In the United States, nearly one in 10 adults (aged ≥20 years) are suffering from CVD (mainly coronary heart disease and stroke) [2], and the annual direct and indirect costs due to CVD in the United States are estimated to amount to US $378 billion [2]. Although the age-standardized mortality rate attributable to CVD has largely declined over the past decades in the United States, the total health and economic burdens due to CVD remain huge, partly due to population growth and aging [3,4].

Given that a large part of the CVD burden is attributable to a limited number of health and behavioral factors, the American Heart Association (AHA) promoted 7 cardiovascular health (CVH) metrics (also known as the Life’s Simple 7 [LS7]) in 2010 [5], including 4 behavioral factors (no smoking, sufficient physical activity [PA], healthy diet, and having a normal BMI), and 3 health factors (normal levels of blood lipids, blood pressure [BP], and blood glucose). Each of these 7 metrics was categorized by scores of 0, 1, and 2 points to represent a poor, intermediate, and ideal health status, respectively. The overall CVH score in the LS7 was calculated as the sum of the scores of these 7 metrics, which can range from 0 (worst health) to 14 points (optimal health). Unfortunately, the prevalence of people with ideal CVH (ie, with high LS7 score) has been consistently extremely low (eg, <1% have an optimal score of 14 points) [2].

The LS7 was widely used in the past decade and had played an important role in promoting CVH in the United States and around the world. However, there are some limitations to the LS7 [6]. First, the categorization (poor, intermediate, and ideal) of the LS7 components inherently reduces a precise assessment of the risk factors that all actually have a graded relation with CVD outcomes. Second, the definitions of poor, intermediate, and ideal categories for each component are arbitrary (eg, PA duration from 1 minute to 149 minutes per week are all categorized in the same intermediate category). Third, several social or behavioral CVD risk factors such as psychological factors and sleep are not included in the LS7. Fourth, some of the LS7 metrics are not assessed comprehensively. For example, the LS7 diet metric was assessed by the sole intake of 4 foods and nutrients (ie, fruits and vegetables, fish, whole grains, sweetened beverages, and sodium).

The newly released Life’s Essential 8 (LE8) metrics proposed by the AHA [6] addressed several of the limitations of LS7 (Table S1 in Multimedia Appendix 1). For example, some score categories have been further defined—sleep health has been added as an eighth metric, the definition of a healthy diet has been expanded, the use of inhaled nicotine-delivery system and secondhand smoke exposure have been considered in addition to combustible cigarette use, hemoglobin A_1c_ level has been added in addition to fasting glucose, and non–high-density lipoprotein cholesterol is used rather than total cholesterol to assess blood lipids [6]. One recent study reported that 47.3% of young individuals who were evaluated as having ideal CVH by the LS7 were reclassified into the low CVH category by the LE8 [7], suggesting that the new LE8 can reduce the misclassification of CVH status. However, although the AHA acknowledged the importance of psychological health and well-being and strongly encouraged more routine assessment and intervention in clinical settings [6], these factors are also not considered in the LE8 as obligatory indicators.

The new CVH score defined by the LE8 has recently been shown to be inversely associated with the risk of all-cause and CVD mortality [8-10]. One study based on 23,110 adults in the United States indicated that every 10-score increase in overall CVH score could decrease the risk of all-cause mortality by 14% and CVD mortality by 19% [8]. Understanding the secular trends of overall CVH and its components is useful to inform and guide targeted health care and public health policies by the US government and other relevant organizations [11]. Therefore, based on the newly published CVH concept of the LE8, we examined the secular trends in overall CVH and its each component among the US adult population based on 7 serial nationally representative cross-sectional surveys conducted between 2005-2006 (which we refer to as “2005” hereafter) and 2017-2018 (which we refer to as “2018”).

## Methods

### Study Population

Data were obtained from the National Health and Nutrition Examination Survey (NHANES), which consists of serial cross-sectional surveys conducted by the National Center for Health Statistic of the US Centers for Disease Control and Prevention to evaluate the health and nutritional status in US adults and children. Participants were selected using a multistage cluster probability sampling method, and all eligible participants were invited to complete a household interview and a physical examination. The questionnaire includes information on demographic, socioeconomic, lifestyle, and other health-related variables, and the physical examination consists of anthropometric and biological measurements. Data from the NHANES are publicly available [12]. The NHANES began in 1999-2000, and subsequent surveys were carried out every 2 years. In this study, we used the data from the 7 survey waves that were conducted from 2005 to 2018 since information on sleep health (1 component of the LE8) was initially collected since 2005. A total of 39,749 adults aged ≥20 years were initially included. We excluded individuals aged >79 years (n=2776), those with missing values on CVH metrics (total of 10,355, including 7996 due to missing data on diet [missing values on all two 24-hour dietary recalls: n=3876; missing on a 24-hour dietary recall: n=4120]) or demographic variables (n=2037), pregnant women (n=481), women who were breastfeeding (n=198), or individuals with self-reported CVD history (n=2235; Figure S1 in Multimedia Appendix 1). A total of 21,667 adults aged 20-79 years were included in this analysis.

We performed analyses in consideration of several demographic characteristics: age, sex, race or ethnicity, educational level, marital status, and household income. We classified participants into three age groups: 20-39, 40-64, and 65-79 years [13]. Self-reported race or ethnicity was classified as Hispanic, non-Hispanic White, non-Hispanic Black, and “Other” (including mainly Asian participants). To characterize an individual’s socioeconomic status, we used the ratio of family income to poverty by dividing family income by the federal poverty threshold for the survey year, adjusting for household size, categorized as <1.30 (low income), 1.30-2.99 (middle income), and ≥3.00 (high income) [14]. Educational level was divided into 4 groups: <high school graduate, high school graduate, some college or associate degree, and college graduate or above. Marital status was categorized as married, divorced/separated/widowed, and unmarried/cohabitation status.

### Ethics Approval

The NHANES was approved by the National Center for Health Statistics Research Ethics Review Board. Written informed consent was obtained from all participants. This study was exempted from ethical approval by the institutional review board of Shandong University given the use of deidentified and open access data.

### Quantification of CVH

The LE8 score is based on 4 “behavioral factors” (diet, PA, smoking, and sleep health) and 4 “health factors” (BMI, blood lipids, blood glucose, and BP). Diet quality was assessed using the Healthy Eating Index 2015 (HEI-2015) based on data from 2 interviewer-administered 24-hour dietary recalls. Diet was assessed using the “Food Patterns Equivalents Database 14” from the US Department of Agriculture [15]. PA was assessed through self-reported frequency and duration of moderate (resulting in light sweating or a small increase in breathing or heart rate) and vigorous activity (resulting in heavy sweating or a large increase in breathing or heart rate) over the past 30 days. The PA duration was calculated by the frequency of PA in a week multiplied by the duration of PA each time. Nicotine exposure was assessed based on self-reported consumption of combustible cigarettes and use of e-cigarettes and other tobacco products in the previous 30 days, as well as self-reported secondhand smoke exposure in a participant’s household. Sleep health was assessed from the question on usual sleep duration per day. Height and weight were objectively measured using a stadiometer and digital weight scale, respectively, and BMI was calculated as weight (kg)/height squared (m^2^). BP was measured for three consecutive times after 5 minutes of seated rest, and the average of the second and third readings was used (or the average of the first and second measurements for participants who only had 2 readings or using the first measurement for participants who only had 1 reading). Information on the use of antihypertensive drugs was obtained from the interviewed questionnaire at home. Blood samples were collected at a mobile examination center and sent to a central laboratory for the assessment of blood lipids, fasting glucose, and hemoglobin A_1c_. Data on the use of lipid-lowering medications, insulin, or oral hypoglycemic agents were obtained from the interviewed questionnaire at home. Changes for some biochemical indexes in measurement methods and used instruments over time were adjusted according to the official recommendation to make them comparable across different survey years [16]. The 8 behavioral and health factors and the thresholds used to allocate scores for each component in detail are shown in Table S2 in Multimedia Appendix 1. Scores for each component ranged from 0 to 100 points according to the AHA scoring algorithm [6], with a higher score meaning a better health status. The overall CVH score was calculated as the mean of the sum of all 8 metrics and similarly ranged from 0 (if the mean score of all components was 0) to 100 (optimal CVH). In this study, scores for the overall CVH and individual components were categorized into poor (0-50 points), intermediate (50-79 points), and high (80-100 points) status according to the AHA recommendation [6].

### Statistical Analysis

Differences in percentages of demographic characteristics across the 7 survey waves were assessed with the chi-square test. We calculated the age-standardized mean scores and their 95% CI for the overall CVH score and for each of the 8 CVH components. We performed stratified analysis by sex, age group, educational level, marital status, race or ethnicity, and family income category. We used linear regression model to estimate the linear trends between 2005 and 2018 with adjustments for sex, age group, educational level, marital status, and family income category if appropriate. Data were standardized for age using a direct standardization method based on the age distribution of the 2018 US population (20-39 years: 38.6%; 40-64 years: 44.6%; 65-79 years: 16.8%) [17]. Since oversampling was done in some particular subgroups of the total population in the NHANES to increase the reliability and precision of health indicators in the specific population, the appropriate sample weights, as well as strata and primary sampling units provided by the NHANES were used to make the data national representative of the US population. All statistical analyses were performed using Stata software (version 16.0, Stata Corp), and 2-sided *P* values of <.05 were considered statistically significant.

## Results

A total of 21,667 participants aged 20-79 years were included in this study. The distributions of sex, age group, race or ethnicity, marital status, and family income did not differ across the survey waves. Participants with an educational level lower than high school decreased from 13.9% in 2005 to 8.6% in 2018 (*P*=.01; Table 1).

**Figure 1:**
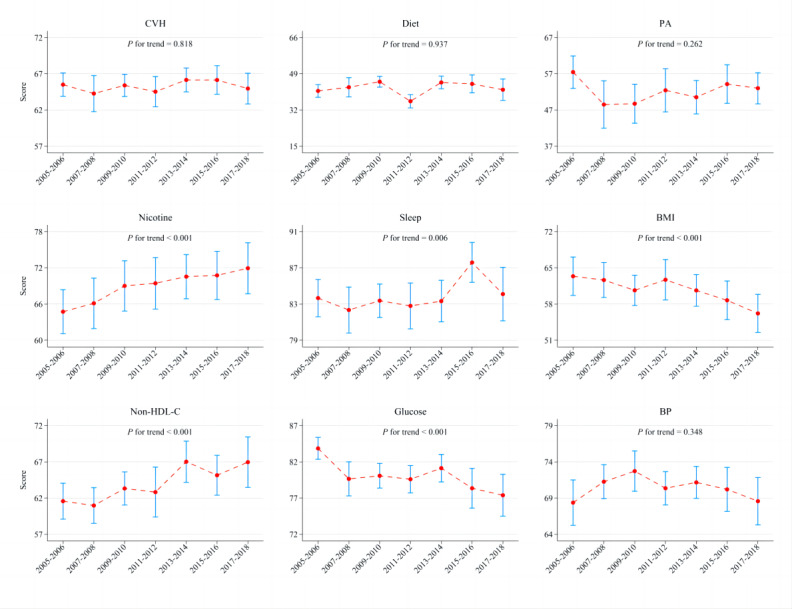
Trends in age-standardized mean score (95% CI) of overall cardiovascular health and its 8 components based on the Life’s Essential 8 in US adults from 2005-2006 to 2017-2018. BP: blood pressure; CVH: cardiovascular health; non–HDL-C: non–high-density lipoprotein cholesterol; PA: physical activity.

**Figure 2:**
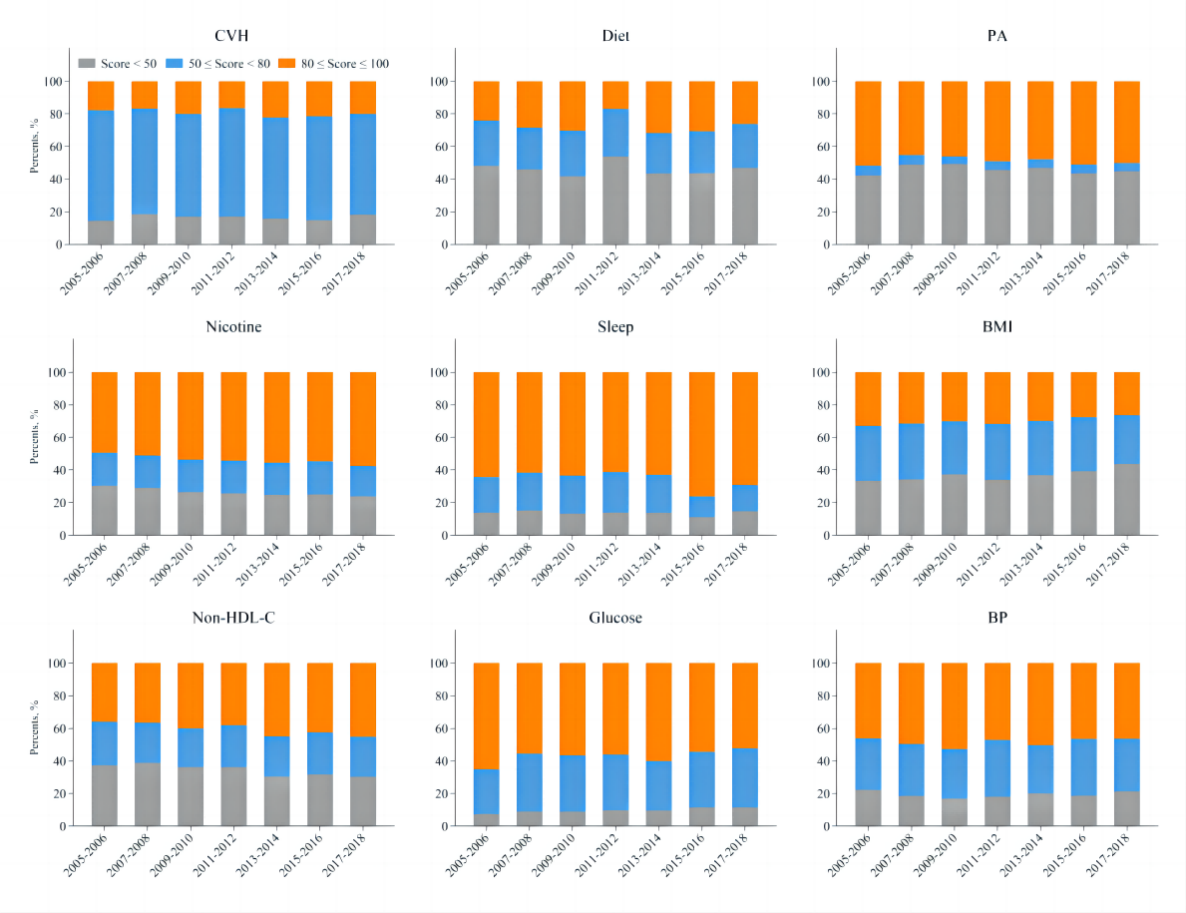
Trends in distributions of age-standardized scores categories of low (<50 points), median (50-79 points), and high (80-100 points) status of overall cardiovascular health and its 8 components based on the Life’s Essential 8 in US adults from 2005-2006 to 2017-2018 (higher score denotes better CVH). BP: blood pressure; CVH: cardiovascular health; non–HDL-C: non–high-density lipoprotein cholesterol; PA: physical activity.

The age-standardized mean overall CVH score changed from 65.5 (95% CI 63.9-67.1) in 2005 to 65.0 (95% CI 62.8-67.1) in 2018 (Figure 1), but the result was not statistically significant (*P* for trend=.82), as well as in almost all subgroups (Table 2). Trends in crude overall CVH score over time showed a similar pattern (Table S3 in Multimedia Appendix 1). Adults with age-standardized high (≥80 points), intermediate (50-79 points), and low (<50 points) overall CVH scores accounted for about 20%, 65%, and 15% in the US population, respectively, in each survey wave (Figure 2). The results were similar when based on the crude proportion (Figure S2 in Multimedia Appendix 1). The overall CVH score significantly worsened between 2005 and 2018 in older, Hispanic, and non-Hispanic White individuals, but no difference was found in other covariate categories.

The age-standardized mean scores of diets, PA, and BP did not significantly change from 2005 to 2018 (Figure 1), as well as in almost all subgroups (Table S4 in Multimedia Appendix 1). The age-standardized mean score of PA fluctuated over time. The age-standardized mean scores improved for nicotine exposure (64.7, 95% CI 61.1-68.4 to 71.9, 95% CI 67.7-76.2), sleep health (83.7, 95% CI 81.6-85.7 to 84.1, 95% CI 81.2-87.1), and blood lipids (61.6, 95% CI 59.1-64.0 to 67.0, 95% CI 63.5-70.4) from 2005 to 2018. However, the age-standardized mean scores significantly decreased for BMI (63.4, 95% CI 59.7-67.1 to 56.2, 95% CI 52.5-59.9) and blood glucose (83.9, 95% CI 82.4-85.4 to 77.4, 95% CI 74.5-80.3; Figure 1, Table S4 in Multimedia Appendix 1). The results were similar within subgroups of sex, age, race or ethnicity, marital status, and family income (Table S4 in Multimedia Appendix 1). Similar trends were found based on the crude scores of the 8 metrics (Table S5 in Multimedia Appendix 1). Among all 8 metrics, blood glucose, sleep health, and nicotine exposure had the largest proportion (>50%) of high score (≥80 points); PA and diets had the largest proportion (40%-50%) of the low score (<50 points) over time (Figure 2). The results were similar when based on crude proportions (Figure S2 in Multimedia Appendix 1). Distributions of scores categories for 8 individual CVH metrics according to the specific scoring algorithm in detail are shown in Figure S3 in Multimedia Appendix 1 (aged-standardized proportion) and Figure S4 in Multimedia Appendix 1 (crude proportion).

By 2018, Americans who were female (66.9, 95% CI 63.9-70.0), young adults aged 20-34 years (69.8, 95% CI 67.2-72.4), non-Hispanic White (65.9, 95% CI 63.1-68.6), or in the “Other” category (including mainly Asian individuals: 66.4, 95% CI 62.9-70.0) had (or tended to had) higher (better) age-standardized mean LE8 scores of overall CVH than those who were male (63.0, 95% CI 60.8-65.2, older adults aged 65-79 years (61.1, 95% CI 59.4-62.9) and non-Hispanic Black (60.1, 95% CI 57.8-62.5), and the age-standardized mean LE8 scores of overall CVH were also much higher in Americans with higher educational level (73.1, 95% CI 70.0-76.1) versus lower educational level (57.1, 95% CI 53.5-60.6), in those who were married (66.3, 95% CI 64.2-68.5) versus those who were unmarried (60.4, 95% CI 56.6-64.2), and in those with higher family income (68.3, 95% CI 65.7-71.0) versus those with lower family income (59.0, 95% CI 55.4-62.6; Table 2).

**Table 1:** Characteristics of the US adult National Health and Nutrition Examination surveys, 2005-2018.

Characteristics		2005-2006 (n=2791)	2007-2008 (n=3250)	2009-2010 (n=3514)	2011-2012 (n=3113)	2013-2014 (n=3292)	2015-2016 (n=2942)	2017-2018 (n=2765)	*P* value^a^
**Sex, %**									.49
	Male	48.5	46.5	48.3	49.7	49.5	49.5	48.5	
	Female	51.5	53.5	51.7	50.3	50.5	50.5	51.5	
**Age group (** **years),** **%**									.19
	20-39	39.5	40.7	38.9	39.9	39.4	38.9	39.7	
	40-64	48.2	48.7	49.6	49.2	48.3	46.5	45.0	
	65-79	12.3	10.6	11.5	10.9	12.3	14.6	15.3	
**Race or ethnicity, %**									.36
	Hispanic	10.5	13.5	13.5	14.5	15.0	15.0	15.6	
	Non-Hispanic White	73.6	71.6	70.2	67.5	65.4	66.3	63.2	
	Non-Hispanic Black	10.9	10.1	10.3	10.7	10.9	9.4	10.4	
	Other	5.0	4.8	6.0	7.3	8.7	9.3	10.8	
**Educational level, %**									.01
	<High school graduate	13.9	18.3	15.8	13.2	12.5	11.0	8.6	
	High school graduate	24.3	24.0	22.4	19.6	20.3	21.2	27.0	
	Some college or associate degree	32.2	29.5	31.5	32.8	34.2	33.5	31.7	
	College graduate or above	29.6	28.2	30.3	34.4	33.0	34.3	32.7	
**Marital status, %**									.47
	Married	59.0	56.0	57.6	53.2	56.6	55.7	53.6	
	Divorced/separated/widowed	16.8	16.5	14.9	15.8	16.7	15.1	16.6	
	Unmarried/cohabitation	24.2	27.5	27.5	31.0	26.7	29.2	29.8	
**Ratio of family income to poverty,^b^ %**									.11
	<1.30	14.6	19.3	21.0	22.7	23.3	18.5	19.4	
	1.30-2.99	27.4	28.4	26.6	27.1	24.4	30.6	26.5	
	≥3.00	58.0	52.3	52.4	50.2	52.3	50.9	54.1	

^a^Differences in percentages of demographic characteristics across the 7 survey waves were assessed with the chi-square test.

^b^The ratio of family income to the federal poverty threshold, which was adjusted for household size.

**Table 2:** Trends in the age-standardized mean score (95% CI) of total cardiovascular health in US adults, overall and by sex, age, race and ethnicity, educational level, marital status, family income to poverty ratio category, 2005-2018.^a^

Characteristics		2005-2006	2007-2008	2009-2010	2011-2012	2013-2014	2015-2016	2017-2018	*P* for trend^b^
Overall		65.5 (63.9-67.1)	64.3 (61.8-66.8)	65.4 (63.9-66.9)	64.5 (62.4-66.6)	66.2 (64.5-67.8)	66.1 (64.2-68.1)	65.0 (62.8-67.1)	.82
**Sex**									
	Male	63.5 (61.5-65.5)	62.2 (59.7-64.7)	63.4 (61.2-65.5)	62.6 (60.1-65.0)	64.6 (62.4-66.8)	64.2 (61.8-66.6)	63.0 (60.8-65.2)	.77
	Female	67.6 (65.6-69.5)	66.1 (63.1-69.1)	67.3 (65.7-69.0)	66.5 (64.0-68.9)	67.7 (65.7-69.7)	68.2 (65.9-70.5)	66.9 (63.9-70.0)	.46
**Age group (years)**									
	20-39	68.9 (67.8-69.9)	67.8 (65.6-70.0)	69.4 (67.4-71.5)	69.7 (67.6-71.8)	69.7 (67.6-71.7)	70.0 (68.5-71.6)	69.8 (67.2-72.4)	.49
	40-64	63.5 (61.7-65.3)	62.1 (59.4-64.8)	63.5 (62.4-64.5)	61.4 (59.3-63.5)	64.0 (62.7-65.4)	63.5 (61.3-65.6)	62.2 (60.4-64.1)	.94
	65-79	63.2 (60.8-65.5)	62.0 (59.4-64.5)	61.2 (59.6-62.8)	61.1 (59.0-63.1)	63.8 (62.3-65.2)	64.3 (61.9-66.7)	61.1 (59.4-62.9)	.02
**Race or ethnicity**									
	Hispanic	64.5 (62.2-66.7)	62.7 (60.9-64.4)	61.9 (59.3-64.6)	61.9 (59.6-64.2)	64.7 (62.1-67.3)	62.9 (61.1-64.6)	63.3 (60.7-66.0)	.02
	Non-Hispanic White	66.4 (64.2-68.6)	64.9 (61.3-68.6)	66.7 (64.8-68.6)	65.3 (62.6-68.0)	66.8 (64.7-68.9)	67.4 (65.2-69.6)	65.9 (63.1-68.6)	.007
	Non-Hispanic Black	59.7 (57.1-62.4)	59.4 (56.7-62.1)	58.1 (55.5-60.8)	60.0 (57.3-62.7)	60.9 (58.9-62.9)	60.0 (56.8-63.2)	60.1 (57.8-62.5)	.70
	Other (including mainly Asian individuals)	65.7 (58.3-73.1)	67.6 (61.4-73.8)	68.7 (63.9-73.6)	68.8 (65.4-72.3)	69.5 (65.3-73.7)	68.2 (63.8-72.5)	66.4 (62.9-70.0)	.93
**Educational level**									
	<High school graduate	59.1 (56.3-62.0)	57.1 (54.2-60.0)	58.0 (55.6-60.4)	56.3 (53.1-59.6)	59.4 (57.0-61.8)	57.7 (54.2-61.2)	57.1(53.5-60.6)	.36
	High school graduate	60.8 (58.6-63.0)	60.6 (57.7-63.6)	59.8 (57.1-62.5)	60.0 (55.9-64.0)	59.9 (57.1-62.7)	59.9 (57.0-62.8)	61.4 (58.5-64.3)	.75
	Some college or associate degree	65.1 (62.7-67.5)	64.2 (61.6-66.9)	64.5 (62.7-66.3)	63.2 (60.9-65.5)	64.7 (62.0-67.5)	65.4 (62.5-68.3)	62.0 (59.8-64.1)	.40
	College graduate or above	72.7 (70.0-75.4)	72.2 (69.4-74.9)	74.3 (71.8-76.9)	71.4 (68.9-74.0)	73.9 (71.7-76.2)	73.5 (71.2-75.9)	73.1 (70.0-76.1)	.57
**Marital status**									
	Married	66.0 (64.0-68.1)	65.0 (62.1-68.0)	66.2 (64.2-68.1)	66.0 (63.6-68.3)	67.5 (65.1-69.9)	67.2(64.9-69.5)	66.3 (64.2-68.5)	.94
	Divorced/separated/widowed	63.5 (60.1-66.8)	59.2 (55.0-63.5)	62.6 (58.9-66.2)	60.3 (57.0-63.7)	62.5 (60.1-64.9)	60.9(56.3-65.4)	63.1 (60.1-66.2)	.74
	Unmarried/cohabitation	64.8 (62.1-67.5)	63.6 (60.6-66.5)	63.0 (59.3-66.6)	62.4 (58.3-66.6)	65.6 (61.3-69.9)	64.3(61.1-67.5)	60.4 (56.6-64.2)	.31
**Ratio of family income to poverty**									
	<1.30	59.7 (57.1-62.4)	58.4 (54.7-62.1)	58.7 (56.3-61.1)	58.6 (54.9-62.4)	60.2 (57.6-62.7)	60.0 (57.4-62.5)	59.0 (55.4-62.6)	.81
	1.30-2.99	62.2 (59.9-64.6)	62.7 (60.3-65.1)	63.2 (61.0-65.3)	62.9 (60.5-65.3)	62.5 (60.2-64.9)	63.3 (60.6-66.1)	62.4 (59.6-65.2)	.66
	≥3.00	68.4 (66.4-70.5)	67.3 (64.2-70.4)	69.0 (66.8-71.1)	68.0 (65.0-71.0)	70.2 (68.0-72.3)	69.6 (67.3-71.8)	68.3 (65.7-71.0)	.88

^a^Higher score denotes better cardiovascular health.

^b^Linear trends were examined using linear regression model, with adjustment for sex, age, race or ethnicity, educational level, marital status, and the ratio of family income to poverty.

## Discussion

### Principal Findings

To our knowledge, this study is the first to examine the secular trends in CVH among US adults based on the new LE8 scoring algorithm proposed by the AHA. Using the 7 cross-sectional surveys between 2005 and 2018, the overall CVH score did not significantly change over time. However, the overall CVH status significantly worsened between 2005 and 2018 in older adults, Hispanic participants, and non-Hispanic White participants. There was no significant change over time for 3 CVH components (diet, PA, and BP) but 3 components (nicotine exposure, sleep health, and blood lipids) significantly improved, whereas 2 components (BMI and blood glucose) significantly, and markedly, worsened over time.

Although a number of public health and health care policies and programs have been implemented to prevent CVD since 2010 [18-20], the decline in age-standardized CVD mortality rate has slowed down or even stagnated considerably in recent years among US adults [2,21]. For example, the age-standardized mortality rate for heart disease declined between 1999 and 2010 by 8.3%, but only by 1.7% between 2010 and 2017 [21]. One important reason for this deceleration of the CVD decline may be that some CVD risk factors have not significantly improved or even deteriorated. This is consistent with other studies, which showed that the mean number of ideal CVH metrics based on the LS7 had not significantly changed in the past years [22,23]. For example, the Behavioral Risk Factor Surveillance System data showed a nonsignificant change in the mean number of ideal CVH metrics from 3.89 in 2011 to 3.96 in 2017 [22]. Another NHANES study among US adults showed that the mean number of ideal CVH components did not significantly change from 2007 to 2018 in both female participants (4.40 to 4.48) and male participants (3.97 to 3.93) [23]. In addition, previous studies among adults based on the LS7 also showed that there was no significant improvement in CVH among US adults [24,25]. Results of our study, which are based on the new LE8 scoring algorithm, are consistent with no change in CVH reported by the abovementioned studies based on the LS7, highlighting that there is still a long way to go to improve CVH among US adults.

The stable trends in the overall CVH score may mask disparities in trends in CVH components. We found that the mean scores of 4 CVH metrics including nicotine exposure (increase), BMI (decrease), non–high-density lipoprotein cholesterol (increase), and blood glucose (decrease) changed over time but not all in the same direction, and some (diet) did not change over time. Unhealthy diet is a major risk factor for CVD [26], but unfortunately, the dietary component gathered the lowest mean score [24]. Despite some improvement in some dietary components, the overall dietary quality did not significantly improve [27]. This emphasizes the need for interventions on those dietary components that tend to decline or do not improve over time. Actually, a number of interventions have already been implemented during the past years in the United States [28]. The smoking rate decreased from 24.8% in 1999-2000 to 18.1% in 2017-2018 [29], and this translated into improved mean LS7 scores of smoking from 1.53 in 2007-2010 to 1.60 in 2015-2018 [24], as well as in our study. Data from the NHANES showed that mean BMI increased from 28.0 kg/m^2^ in 1999-2000 to 29.8 kg/m^2^ in 2017-2018 [29], with the corresponding obesity prevalence dramatically increasing from 27.5% to 43.0% [30]. He et al [29] reported that the mean total cholesterol decreased from 203.3 mg/dL in 1999-2000 to 188.5 mg/dL in 2017-2018, which is consistent with our finding that the LE8 blood lipid component improved over time. Previous studies showed that the estimated prevalence of diabetes increased from 9.8% in 1999-2000 to 14.3% in 2017-2018 [31], consistent with the decreasing LS7 mean score of blood glucose from 1.42 in 2007-2010 to 1.29 in 2015-2018 [24], as well as the decreasing LE8 mean score observed in our study.

However, there were also several inconsistent findings in our study compared with previous studies regarding the secular trends in 2 CVH components (PA and BP) [24,32,33]. It is reported that the estimated prevalence of meeting the PA guideline target increased from 26.0% in 1998 to 37.4% in 2018 [32]. However, the overall mean score of PA in our study still remained unchanged and at a low level. As for hypertension, the prevalence as defined by the 2017 American College of Cardiology/AHA decreased (suggesting an improvement) from 48.1% in 1999-2000 to 44.1% in 2015-2016 [33]. However, another study reported that the mean BP score based on the LS7 slightly decreased (suggesting a deterioration) from 1.28 in 2007-2010 to 1.24 in 2015-2018 [24]. Inconsistent with the above 2 studies, we did not observe a significant change in the mean LE8 component score of BP between 2005 and 2018. In addition, the national data showed that more than half of the US population did not reach the sufficient sleep duration (20-64 years: 7-9 hours per day, 65-79 years: 7-8 hours per day) recommended by the National Sleep Foundation [34]. Inappropriate sleep duration (<6 hours or ≥9 hours) has been associated with an increased risk of CVD and related mortality [35]. Although previous studies reported a persistent deterioration of sleep quality in the US population from 2005 to 2018 [36,37], we observed an opposite trend with a significant increase (improvement) in the mean LE8 component score of sleep health from 2005 to 2018. These disparities might be partly due to methodological issues in assessing sleep quality across studies.

Consistent with previous studies [13,14], the overall LE8 score of CVH and its components differed markedly across the subgroups of sex, age, race or ethnicity, and socioeconomic status, with individuals who were female, younger, non-Hispanic White, and those with better socioeconomic status being more likely to have a better CVH. Although trends in the CVH metrics over time in these subgroups were mostly consistent with those in the overall population, there are some interesting findings. First, non-Hispanic White participants and the “Other” group (including mainly Asian participants) had a higher overall CVH score than non-Hispanic Black participants, but the difference decreased over time, partly due to deterioration in CVH over time among non-Hispanic White participants. The deterioration of 2 CVH components (diet and PA) among non-Hispanic White participants may be the reason for the worsening trend in overall CVH score in this subgroup. This highlights the need to consider LE8 trends according to racial or ethnic groups when designing CVD prevention and control policy and programs. Second, none of the CVH metrics significantly improved among older participants (65-79 years) over time. CVH usually markedly decreases with age [38], largely driven by the strong relation among hypertension, dyslipidemia, and type 2 diabetes with age. The CVH in older adults is a main challenge, as most hard CVD outcomes develop at an older age. In general, the less ideal CVH at all ages found in this study stresses the need for life course approaches to CVH and the need for high-risk individual-level approaches (clinical care) at middle and older ages. Third, the mean LE8 component of BMI (obesity) markedly deteriorated in all subgroups. This trend is particularly worrying, given the major role of adiposity on glucose metabolism, including insulin resistance, hypertension, and more generally poor CVH [39,40]. This highlights the need to strengthen preventive and medical approaches to weight gain prevention and control, including a broader use of novel effective clinical approaches [41]. Fourth, socioeconomic status such as educational level and family income are important social determinants of CVD [42]. Consistent with abundant literature on this topic, our results show that participants with higher socioeconomic status had much better CVH and the CVH gap (as assessed with the LE8 metrics) between individuals with low versus high socioeconomic status did not decrease over time, highlighting the need to further address social disparities in CVD prevention and control.

### Strengths and Limitations

Our study is the first to examine the secular trends in CVH from 2005 to 2018 among US adults based on the nationally representative NHANES data using the new AHA LE8 metrics. However, several limitations should be mentioned. First, our study was based on data from 7 surveys over a rather short time period (<15 years). Second, data on diet, PA, nicotine exposure, and sleep health were obtained by self-report, which may be inaccurate due to recall and social desirability biases. Third, the 8 behavioral and health components are given equal weight (implying the same predictive performance) in calculating the LE8 overall CVH score. Fourth, although the 8 components constitute major or important risk/preventive factors for CVH, a number of other factors (eg, familial history/genetics, other conditions, or comorbidities) can largely alter CVD risk, particularly at the individual level. Fifth, missing values on relevant variables, especially for dietary variables, might have affected the representativeness of the study population. However, the inclusion of data on one 24-hour dietary recall showed similar results (age-standardized score did not significantly change from 40.46 in 2005-2006 to 40.47 in 2017-2018, *P* for trend=.77) with the primary ones.

### Conclusions

In summary, based on the LE8 metrics, the overall CVH score did not significantly change from 2005 to 2018 among US adults. Very few individuals had CVH scores in good or optimal ranges, implying a large scope for improvement for most adults. Three CVH components remained unchanged over time (diet, PA, and BP), 3 improved (nicotine exposure, sleep health, and blood lipids), and 2 worsened (BMI and blood glucose), suggesting avenues for improving CVH status among US adults.

## Appendix

Multimedia Appendix 1 Supplementary figures and tables.

## Abbreviations

AHA: American Heart Association
BP: blood pressure
CVD: cardiovascular disease
CVH: cardiovascular health
LE8: Life’s Essential 8
LS7: Life’s Simple 7
NHANES: National Health and Nutrition Examination Survey
PA: physical activity

## Data Availability

The data sets generated and analyzed during this study are publicly available on the US Centers for Disease Control and Prevention website [12].
