# Supplementary material for: Using the New “Life’s Essential 8” Metrics to Evaluate Trends in Cardiovascular Health Among US Adults From 2005 to 2018: Analysis of Serial Cross-sectional Studies
Source: JMIR Public Health Surveill. 2023 May 8;9:e45521. doi: 10.2196/45521 (PMC10203917; doi:10.2196/45521)
Supplement: Multimedia Appendix 1 [file publichealth_v9i1e45521_app1.doc]

**Figure S1.** Flowchart for inclusion and exclusion of study participant





**Figure S2.** Trends in distributions of crude scores categories of low (<50 points), median (50-79 points), and high (80-100 points) status of overall cardiovascular health and individual components based on Life’s Essential 8 in US adults from 2005-2006 to 2017-2018 (higher score denotes better cardiovascular health)





**Figure S3.** Trends in distributions of age-standardized score categories of each cardiovascular health component based on Life’s Essential 8 in US adults from 2005-2006 to 2017-2018 (see Table S2 for explanations of score categories; higher score denotes better cardiovascular health)





**Figure S4.** Trends in distributions of crude score categories of each cardiovascular health component based on Life’s Essential 8 in US adults from 2005-2006 to 2017-2018 (see Table S2 for explanations of score categories; higher score denotes better cardiovascular health)

**Table S1** Comparisons between Life’ Simple 7 and Life’ Essential 8

| Domains | Life’ Simple 7 | Life’ Essential 8 |
| --- | --- | --- |
| Components | Dietary quality, physical activity, nicotine exposure, BMI, fasting blood glucose, total cholesterol, blood pressure | Dietary quality, physical activity, nicotine exposure, BMI, fasting blood glucose, total cholesterol, blood pressure, sleep health |
| Score categorizations | Three groups for each metric (low, intermediate, high) | Five groups or more for each metric (e.g, blood lipid was categorized into five groups: 0, 20, 40, 60, and 100 points) |
| Score range | 0-2 points for each CVH metric; 0-14 points for overall CVH | 0-100 points for overall CVH and each metric |
| Definition of specific metric | Diet: diet quality was assessed by five components (fruits and vegetables, fish, fiber-rich whole grains, sodium, sugar-sweetened beverages) | Diet: diet quality was assessed by the intake of a HEI-2015-style or Mediterranean eating pattern |
|  | Nicotine exposure: secondhand smoke exposure was not included | Nicotine exposure: secondhand smoke exposure was included |
|  | Blood lipids: total lipoprotein cholesterol as the metric | Blood lipids: non-high-density lipoprotein cholesterol as the metric |
|  | Blood glucose: hemoglobin A1c was not included | Blood glucose: hemoglobin A1c was included |

**Table S2**. Scoring algorithm used for defining cardiovascular health, according to the American Heart Association’s Life’s Essential 8 score

| **Domain** | **CVH metric** | **Measurement** | **Quantification of CVH metric** |
| --- | --- | --- | --- |
| **Behavioral factors** | **Diet** | Self-reported daily intake of a HEI-2015-style eating pattern | Points Quantile  100 ≥95th (ideal)  80 75 – 94 th  50 50 – 74 th  25 25 – 49 th  0 1 – 24th (least ideal) |
|  | **Physical**  **activity** | Self-reported minutes of moderate or vigorous physical activity per week based on NHANES PAQ-K questionnaire. | Points Minutes  100 ≥150  90 120 – 149  80 90 – 119  60 60 – 89  40 30 – 59  20 1 – 29  0 0 |
|  | **Nicotine**  **exposure** | Self-reported use of cigarettes or inhaled nicotine-delivery system (NDS) based on NHANES SMQ | Points Categories  100 Never smoker  75 Former smoker, quit ≥5 years  50 Former smoker, quit 1 - <5 years  25 Former smoker, quit <1 year, or currently using inhaled NDS  0 Current smoker  Subtract 20 points (unless score is 0) for living with active indoor smoker in home |
|  | **Sleep health** | Self-reported average hours of sleep per night based on responses to the question: “On average, how many hours of sleep do you get per night?” | Points Hours  100 7 – <9  90 9 – <10  70 6 – <7  40 5 – <6 or ≥10  20 4 – <5  0 <4 |
| **Health**  **Factors** | **Body mass index** | Body mass index calculated as weight divided by height squared (kg/m^2^) | Points kg/m^2^  100 <25.0  70 25.0 – 29.9  30 30.0 – 34.9  15 35.0 – 39.9  0 ≥40.0 |
|  | **Blood lipids** | Non-HDL-cholesterol (calculated from fasting or non-fasting total and HDL cholesterol, mg/dl) | Points mg/dl  100 <130  60 130 – 159  40 160 – 189  20 190 – 219  0 ≥220  If drug-treated level, subtract 20 points |
|  | **Blood glucose** | Fasting blood glucose (FBG, mg/dl) or casual hemoglobin A1c (%) | Points Categories  100 No history of diabetes and FBG <100 mg/dl (or HbA1c < 5.7%)  60 No diabetes and FBG 100 – 125 mg/dl (or HbA1c 5.7 – 6.4%) (pre-diabetes)  40 Diabetes with HbA1c <7.0 %  30 Diabetes with HbA1c 7.0 – 7.9 %  20 Diabetes with HbA1c 8.0 – 8.9 %  10 Diabetes with HbA1c 9.0 – 9.9 %  0 Diabetes with HbA1c ≥10.0 % |
|  | **Blood pressure** | Appropriately measured systolic and diastolic blood pressure (mmHg) using appropriately sized blood pressure cuff | Points mmHg  100 <120/<80 (optimal)  75 120 – 129/<80 (elevated)  50 130 – 139 or 80 – 89 (stage 1 hypertension)  25 140 – 159 or 90 – 99  0 ≥160 or ≥100  Subtract 20 points if treated level |

*Note*: This table is adapted from Circulation [6].

**Table S3.** Trends in crude mean scores (95% confidence interval) for total cardiovascular health for US adults, overall and by sex, age, race/ethnicity, education level, marital status, and family income to poverty ratio category, 2005-2018.

| **Characteristics** | 2005-2006 | 2007-2008 | 2009-2010 | 2011-2012 | 2013-2014 | 2015-2016 | 2017-2018 | *P* for trend ^*^ |
| --- | --- | --- | --- | --- | --- | --- | --- | --- |
| **Overall** | 65.6 (64.3-66.8) | 64.4 (62.1-66.7) | 65.5 (64.3-66.8) | 64.7 (62.7-66.6) | 66.2 (6-5.267.22) | 66.2 (64.6-67.7) | 65.1 (63.4-66.7) | .82 |
| **Sex** |  |  |  |  |  |  |  |  |
| Male | 63.6 (62.2-65.0) | 62.4 (60.4-64.4) | 63.5 (61.9-65.2) | 62.9 (60.8-64.9) | 64.8 (6-3.366.28) | 64.3 (62.6-66.0) | 63.2 (61.9-64.5) | .77 |
| Female | 67.4 (65.9-68.9) | 66.2 (63.4-68.9) | 67.4 (66.2-68.6) | 66.4 (64.2-68.6) | 67.6 (6-6.269.04) | 68.0 (66.3-69.6) | 66.8 (64.3-69.3) | .46 |
| **Age group, y** |  |  |  |  |  |  |  |  |
| 20-39 | 68.9 (67.8-69.9) | 67.8 (65.6-70.0) | 69.4 (67.4-71.5) | 69.7 (67.6-71.8) | 69.7 (67.6-71.7) | 70.0 (68.5-71.6) | 69.8 (67.2-72.4) | .49 |
| 40-64 | 63.5 (61.7-65.3) | 62.1 (59.4-64.8) | 63.5 (62.4-64.5) | 61.4 (59.3-63.5) | 64.0 (62.7-65.4) | 63.5 (61.3-65.6) | 62.2 (60.4-64.1) | .94 |
| 65-79 | 63.2 (60.8-65.5) | 62.0 (59.4-64.5) | 61.2 (59.6-62.8) | 61.1 (59.0-63.1) | 63.8 (62.3-65.2) | 64.3 (61.9-66.7) | 61.1 (59.4-62.9) | .02 |
| **Race/Ethnicity** |  |  |  |  |  |  |  |  |
| Hispanic | 65.8 (64.4-67.3) | 64.1 (63.1-65.1) | 63.4 (61.5-65.3) | 63.7 (61.8-65.6) | 66.0 (63.9-68.2) | 63.9 (62.7-65.2) | 64.6 (62.4-66.7) | .02 |
| Non-Hispanic White | 66.3 (64.4-68.1) | 64.9 (61.4-68.3) | 66.6 (65-68.1) | 64.9 (62.5-67.4) | 66.6 (65.4-67.9) | 67.1 (65.5-68.6) | 65.6 (63.5-67.6) | .007 |
| Non-Hispanic Black | 60.3 (58.8-61.8) | 60.1 (58.0-62.3) | 58.7 (56.7-60.8) | 60.6 (59.3-62.0) | 61.2 (60.2-62.1) | 60.6 (58.1-63.1) | 60.9 (59.3-62.5) | .70 |
| Other | 66.1 (62.3-70.0) | 67.0 (62.6-71.3) | 70.1 (66.9-73.3) | 69.9 (67.5-72.3) | 69.7 (66.7-72.7) | 68.9 (65.1-72.7) | 66.8 (64.4-69.1) | .93 |
| **Education level** |  |  |  |  |  |  |  |  |
| <High school graduate | 59.4 (57.6-61.1) | 57.4 (55.4-59.5) | 57.8 (56.3-59.3) | 56.5 (54.3-58.7) | 59.4 (57.6-61.2) | 57.8 (55.1-60.4) | 57.0 (54.9-59.2) | .36 |
| High school graduate | 60.7 (59.6-61.7) | 60.5 (57.8-63.2) | 59.7 (57.6-61.8) | 59.6 (56.2-63.0) | 59.9 (58.2-61.6) | 60.1 (57.4-62.7) | 61.4 (60.0-62.8) | .75 |
| Some college or AA degree | 65.4 (63.6-67.3) | 64.5 (62.7-66.3) | 65.0 (63.6-66.4) | 63.7 (61.5-65.9) | 65.1 (63.7-66.5) | 65.4 (63.6-67.3) | 62.2 (60.6-63.8) | .40 |
| College graduate or above | 72.7 (70.6-74.8) | 72.2 (70.2-74.1) | 74.5 (72.3-76.7) | 71.6 (69.7-73.4) | 73.8 (72.2-75.5) | 73.3 (71.7-75.0) | 73.0 (70.7-75.3) | .57 |
| **Marital status** |  |  |  |  |  |  |  |  |
| Married | 65.8 (64.3-67.4) | 64.7 (62.1-67.3) | 65.9 (64.6-67.2) | 65.6 (63.7-67.5) | 67.0 (65.8-68.2) | 66.8 (65.2-68.5) | 65.7 (64.0-67.3) | .94 |
| Divorced/separated/widowed | 61.9 (60.0-63.7) | 59.3 (56.5-62.0) | 61.5 (59.6-63.3) | 59.1 (56.7-61.5) | 61.1 (60.0-62.1) | 59.7 (57.1-62.4) | 62.0 (60.0-63.9) | .74 |
| Unmarried/cohabitation | 67.5 (66.2-68.9) | 66.9 (64.7-69.1) | 67.0 (64.6-69.5) | 65.9 (63.1-68.8) | 67.8 (65.6-70.1) | 68.2 (66.4-69.9) | 65.7 (62.7-68.7) | .31 |
| **Ratio of family income to poverty** |  |  |  |  |  |  |  |  |
| <1.30 | 60.6 (58.9-62.3) | 59.7 (56.5-62.9) | 60.0 (58.5-61.4) | 60.5 (56.4-64.6) | 61.6 (59.8-63.5) | 61.0 (59.3-62.6) | 60.3 (57.5-63.1) | .81 |
| 1.30-2.99 | 62.8 (61.2-64.3) | 63.1 (60.9-65.2) | 63.6 (62.0-65.1) | 63.1 (61.4-64.7) | 63.1 (61.7-64.6) | 64.4 (62.3-66.4) | 62.7 (60.8-64.6) | .66 |
| ≥3.00 | 68.1 (66.8-69.5) | 66.9 (64.4-69.3) | 68.8 (66.9-70.6) | 67.4 (64.9-69.9) | 69.7 (68.5-70.9) | 69.1 (67.7-70.5) | 67.9 (66.3-69.6) | .88 |

* Linear trends were examined using linear regression, with adjustment for sex, age, race/ethnicity, education level, marital status, and ratio of family income to poverty.

*Note*: higher score denotes better cardiovascular health

**Table S4.** Trends in age-standardized mean scores (95% confidence interval) for individual CVH metrics for US adults, overall and by sex, age, race/ethnicity, education level, marital status and family income to poverty ratio category, 2005-2018

| **Characteristics** | 2005-2006 | 2007-2008 | 2009-2010 | 2011-2012 | 2013-2014 | 2015-2016 | 2017-2018 | *P* for trend^*^ |
| --- | --- | --- | --- | --- | --- | --- | --- | --- |
| **Diet** |  |  |  |  |  |  |  |  |
| **Overall** | 41.0 (38.0-43.9) | 42.7 (38.2-47.2) | 45.3 (42.7-47.9) | 36.1 (33.0-39.2) | 44.9 (42.0-47.9) | 44.3 (40.0-48.5) | 41.5 (36.5-46.6) | .94 |
| **Sex** |  |  |  |  |  |  |  |  |
| Male | 37.1 (33.2-41.0) | 39.5 (35.0-44.0) | 41.1 (37.5-44.8) | 34.1 (30.4-37.8) | 41.0 (37.1-45.0) | 41.3 (35.8-46.7) | 38.6 (33-44.3) | .70 |
| Female | 44.4 (40.8-47.9) | 45.4 (39.6-51.2) | 49.0 (46.1-51.8) | 38.1 (33.5-42.6) | 48.6 (45.1-52.1) | 47.4 (41.8-53.0) | 44.3 (38.7-50) | .58 |
| **Age group, y** |  |  |  |  |  |  |  |  |
| 20-39 | 32.8 (29.8-35.9) | 34.6 (31.1-38.2) | 39.5 (36.5-42.5) | 32.1 (29.6-34.5) | 38.6 (35.4-41.7) | 38.5 (34.1-42.8) | 36.3 (31.4-41.2) | .23 |
| 40-64 | 43.4 (40.8-46.1) | 45.1 (40.1-50.1) | 46.6 (44.3-48.8) | 37.3 (34.7-40.0) | 46.8 (44.5-49.1) | 46.7 (43.1-50.3) | 42.8 (37.9-47.8) | .88 |
| 65-79 | 53.1 (49.2-56.9) | 54.7 (49.3-60.1) | 55.2 (52.9-57.5) | 42.2 (36.3-48.1) | 54.7 (50.3-59.1) | 51.0 (45.3-56.7) | 50.0 (44.4-55.7) | .02 |
| **Race/ethnicity** |  |  |  |  |  |  |  |  |
| Hispanic | 40.8 (35.0-46.5) | 45.9 (41.1-50.8) | 45.0 (40.0-50.1) | 38.9 (33.9-43.9) | 47.5 (40.8-54.3) | 43.3 (38.0-48.5) | 45.3 (38.3-52.2) | .86 |
| Non-Hispanic White | 41.5 (38.1-44.9) | 42.7 (36.6-48.8) | 45.5 (42.4-48.6) | 34.9 (30.8-39.0) | 43.7 (39.8-47.6) | 44.5 (39.4-49.7) | 40.2 (33.6-46.8) | .001 |
| Non-Hispanic Black | 35.0 (30.4-39.6) | 37.2 (31.9-42.6) | 39.2 (33.9-44.6) | 34.5 (29.6-39.4) | 40.1 (35.5-44.6) | 36.9 (31.6-42.2) | 39.0 (32.8-45.2) | .07 |
| Other (including mainly Asians) | 48.5 (37.5-59.4) | 46.5 (36.6-56.5) | 48.4 (40.0-56.7) | 44.8 (37.1-52.4) | 55.2 (47.3-63.2) | 52.7 (43.9-61.5) | 48.4 (41.2-55.6) | .11 |
| **Education level** |  |  |  |  |  |  |  |  |
| <High school graduate | 36.1 (29.8-42.4) | 34.8 (29.0-40.6) | 40.0 (34.6-45.5) | 31.8 (25.7-37.8) | 39.3 (33.3-45.2) | 34.9 (28.6-41.1) | 36.8 (28.4-45.1) | .72 |
| High school graduate | 33.9 (28.6-39.2) | 36.1 (30.9-41.4) | 36.6 (31.6-41.6) | 31.1 (25.9-36.2) | 33.1 (26.9-39.3) | 37.2 (31.1-43.3) | 34.0 (27.7-40.3) | .69 |
| Some college or associate degree | 39.9 (34.8-45.0) | 43.7 (39.0-48.5) | 43.1 (38.6-47.6) | 33.7 (29.0-38.3) | 43.6 (38.5-48.6) | 42.0 (35.8-48.2) | 37.7 (31.1-44.4) | .61 |
| College graduate or above | 49.8 (44.2-55.4) | 52.9 (45.7-60.2) | 56.8 (52.3-61.3) | 43.2 (36.7-49.6) | 55.9 (51.0-60.7) | 54.0 (47.8-60.2) | 53.1 (46.7-59.5) | .50 |
| **Marital status** |  |  |  |  |  |  |  |  |
| Married | 41.2 (38.0-44.5) | 44.0 (39.6-48.4) | 46.8 (43.4-50.3) | 39.0 (35.0-42.9) | 46.4 (42.0-50.9) | 45.8 (41.0-50.6) | 43.3 (37.6-49.0) | .80 |
| Divorced/separated/widowed | 40.8 (33.6-48.0) | 37.4 (28.8-46.0) | 42.4 (34.8-50.1) | 30.4 (25.5-35.3) | 44.3 (37.9-50.8) | 42.0 (34.0-50.0) | 39.9 (33.8-46.1) | .69 |
| Unmarried/cohabitation | 40.1 (33.2-47.0) | 40.0 (31.5-48.6) | 44.1 (38.8-49.5) | 32.5 (26.1-38.9) | 43.3 (37.2-49.4) | 41.4 (34.0-48.8) | 39.7 (29.8-49.5) | .80 |
| **Ratio of family income to poverty** |  |  |  |  |  |  |  |  |
| <1.30 | 36.3 (30.2-42.5) | 38.0 (32.0-44.0) | 39.4 (34.8-43.9) | 31.1 (25.6-36.7) | 38.1 (33.0-43.2) | 37.4 (32.4-42.4) | 37.7 (31.5-43.9) | .55 |
| 1.30-2.99 | 36.6 (30.7-42.5) | 40.3 (33.7-46.9) | 42.1 (36.6-47.7) | 33.0 (28.4-37.6) | 40.4 (34.4-46.4) | 39.2 (34.6-43.8) | 39.0 (32.1-45.9) | .86 |
| ≥3.00 | 44.2 (40.2-48.1) | 45.6 (40.5-50.7) | 48.7 (45.6-51.8) | 39.8 (35.2-44.4) | 49.9 (46.1-53.8) | 49.6 (43.5-55.6) | 44.8 (38.6-50.9) | .79 |
| **Physical activity** |  |  |  |  |  |  |  |  |
| **Overall** | 57.5 (53.0-61.9) | 48.5 (42.0-55.0) | 48.7 (43.3-54.1) | 52.4 (46.5-58.4) | 50.5 (45.9-55.2) | 54.2 (48.8-59.5) | 53.0 (48.7-57.3) | .26 |
| **Sex** |  |  |  |  |  |  |  |  |
| Male | 57.8 (52.3-63.4) | 51.8 (44.7-59.0) | 53.2 (46.4-60.0) | 54.0 (47.3-60.6) | 51.2 (46.3-56.1) | 54.5 (47.7-61.3) | 54.5 (49.0-60.0) | .10 |
| Female | 57.3 (51.5-63.0) | 45.6 (37.4-53.7) | 44.4 (38.7-50.1) | 50.6 (43.0-58.3) | 49.7 (42.2-57.2) | 53.9 (47.2-60.7) | 51.8 (44.9-58.6) | .72 |
| **Age group, y** |  |  |  |  |  |  |  |  |
| 20-39 | 62.5 (58.9-66.2) | 57.6 (52.9-62.3) | 55.2 (48.5-61.9) | 60.8 (55.1-66.4) | 56.2 (50.7-61.6) | 59.5 (54.5-64.5) | 62.3 (57.8-66.7) | .79 |
| 40-64 | 56.3 (52.3-60.3) | 45.1 (36.9-53.3) | 47.2 (42.7-51.8) | 48.9 (43.3-54.5) | 48.7 (45.6-51.7) | 49.5 (44.5-54.5) | 47.3 (43.6-50.9) | .12 |
| 65-79 | 48.9 (41.3-56.5) | 36.7 (30.4-43.1) | 37.8 (33.2-42.4) | 42.7 (35.2-50.3) | 42.6 (35.8-49.4) | 54.2 (47.2-61.1) | 47.1 (41.4-52.7) | .97 |
| **Race/ethnicity** |  |  |  |  |  |  |  |  |
| Hispanic | 48.3 (37.3-59.2) | 35.0 (29.0-40.9) | 36.4 (29.8-43.0) | 39.4 (31.1-47.7) | 45.3 (37.4-53.2) | 42.6 (36.0-49.2) | 46.6 (37.6-55.6) | .45 |
| Non-Hispanic White | 60.1 (54.5-65.6) | 52.0 (41.9-62.0) | 52.4 (45.6-59.3) | 56.6 (49.7-63.6) | 51.6 (45.5-57.8) | 57.9 (51.1-64.8) | 55.3 (49.2-61.4) | .001 |
| Non-Hispanic Black | 49.6 (42.0-57.1) | 40.4 (33.1-47.8) | 39.9 (31.7-48.2) | 45.1 (36.7-53.5) | 45.6 (37.8-53.4) | 45.1 (37.0-53.2) | 46.4 (38.4-54.3) | .86 |
| Other (including mainly Asians) | 57.5 (36.7-78.3) | 50.5 (29.6-71.3) | 46.6 (30.0-63.1) | 51.1 (38.6-63.7) | 59.0 (49.3-68.7) | 52.4 (42.1-62.7) | 53.1 (41.2-65.1) | .14 |
| **Education level** |  |  |  |  |  |  |  |  |
| <High school graduate | 37.6 (26.1-49.1) | 28.7 (20.8-36.5) | 26.4 (19.4-33.4) | 28.0 (19.8-36.2) | 30.4 (23.6-37.3) | 27.9 (19.3-36.4) | 29.6 (18.7-40.5) | .16 |
| High school graduate | 48.3 (40.7-56.0) | 42.9 (33.7-52.1) | 37.3 (30.4-44.2) | 46.0 (33.8-58.3) | 36.2 (29.6-42.8) | 42.9 (33.6-52.2) | 45.3 (37.3-53.3) | .64 |
| Some college or associate degree | 58.0 (51.2-64.8) | 50.8 (42.7-58.8) | 49.6 (42.2-56.9) | 49.9 (42.7-57.2) | 50.5 (42.6-58.3) | 53.9 (43.4-64.4) | 50.0 (43.5-56.5) | .42 |
| College graduate or above | 73.4 (67.0-79.9) | 64.0 (56.0-71.9) | 67.8 (58.9-76.8) | 67.9 (60.7-75.1) | 66.9 (61.2-72.6) | 69.7 (62.7-76.7) | 68.7 (61.3-76.1) | .84 |
| **Marital status** |  |  |  |  |  |  |  |  |
| Married | 56.9 (51.4-62.3) | 49.7 (41.6-57.8) | 47.8 (40.3-55.3) | 53.4 (46.9-59.8) | 51.7 (44.8-58.7) | 56.0 (49.5-62.4) | 54.4 (48.0-60.9) | .71 |
| Divorced/separated/widowed | 60.1 (50.7-69.4) | 38.3 (28.4-48.3) | 49.2 (39.5-59.0) | 48.1 (35.6-60.5) | 48.4 (40.2-56.5) | 45.2 (35.7-54.7) | 50.5 (40.2-60.8) | .36 |
| Unmarried/cohabitation | 55.7 (46.4-65.1) | 49.0 (40.1-57.9) | 43.6 (34.9-52.4) | 49.0 (38.2-59.9) | 51.6 (40.9-62.3) | 51.8 (40.0-63.6) | 45.8 (36.5-55.2) | .14 |
| **Ratio of family income to poverty** |  |  |  |  |  |  |  |  |
| <1.30 | 43.5 (34.4-52.7) | 32.2 (23.6-40.9) | 32.7 (24.2-41.1) | 37.1 (27.7-46.5) | 39.3 (31.1-47.5) | 42.3 (35.3-49.4) | 38.2 (28.5-47.9) | .98 |
| 1.30-2.99 | 50.2 (44.4-56.1) | 45.1 (35.2-55.1) | 41.7 (35.5-48.0) | 52.2 (43.7-60.7) | 44.8 (35.8-53.8) | 45.0 (36.9-53.1) | 45.5 (36.5-54.5) | .14 |
| ≥3.00 | 64.2 (59.2-69.1) | 56.6 (48.0-65.3) | 58.4 (50.9-65.8) | 59.4 (51.5-67.3) | 59.0 (53.3-64.7) | 62.9 (55.6-70.2) | 61.8 (55.1-68.5) | .54 |
| **Nicotine exposure** |  |  |  |  |  |  |  |  |
| **Overall** | 64.7 (61.1-68.4) | 66.1 (61.9-70.3) | 69.0 (64.8-73.2) | 69.4 (65.2-73.7) | 70.5 (66.9-74.2) | 70.8 (66.8-74.7) | 71.9 (67.7-76.2) | <.001 |
| **Sex** |  |  |  |  |  |  |  |  |
| Male | 60.2 (55.1-65.4) | 60.7 (55.4-65.9) | 63.5 (57.9-69.2) | 64.0 (57.8-70.2) | 67.6 (62.6-72.6) | 65.3 (59.9-70.7) | 65.9 (59.7-72.0) | .003 |
| Female | 69.0 (64.6-73.5) | 70.9 (65.8-76.0) | 74.1 (69.2-79.0) | 74.8 (70.0-79.5) | 73.4 (69.0-77.9) | 76.2 (71.3-81.0) | 77.6 (73.0-82.1) | .002 |
| **Age group, y** |  |  |  |  |  |  |  |  |
| 20-39 | 59.5 (55.7-63.3) | 61.0 (55.8-66.2) | 63.4 (57.4-69.4) | 68.0 (63.1-72.9) | 66.0 (61.7-70.2) | 66.6 (63.2-70.1) | 69.4 (65.1-73.8) | .001 |
| 40-64 | 66.0 (62.7-69.3) | 66.2 (62.6-69.8) | 70.0 (66.9-73.1) | 67.8 (64.1-71.6) | 71.0 (67.7-74.3) | 70.7 (66.4-75.0) | 72.1 (67.9-76.2) | .005 |
| 65-79 | 73.3 (69.2-77.4) | 77.6 (74.3-80.9) | 79.3 (76.5-82.0) | 77.0 (72.8-81.2) | 79.8 (76.6-83.0) | 80.4 (75.9-84.8) | 77.3 (73.2-81.4) | .66 |
| **Race/ethnicity** |  |  |  |  |  |  |  |  |
| Hispanic | 74.1 (68.8-79.4) | 72.0 (66.4-77.6) | 73.2 (67.8-78.6) | 75.0 (69.2-80.8) | 78.3 (72.6-84.0) | 75.7 (71.2-80.2) | 77.3 (71.0-83.6) | .01 |
| Non-Hispanic White | 63.1 (57.9-68.4) | 64.5 (58.6-70.3) | 67.8 (62.3-73.3) | 67.6 (62.1-73.2) | 68.3 (62.9-73.7) | 69.4 (64.2-74.6) | 70.5 (64.9-76.2) | .008 |
| Non-Hispanic Black | 62.0 (53.0-70.9) | 63.7 (57.5-69.8) | 64.5 (55.5-73.4) | 66.8 (58.6-75.0) | 65.9 (59.7-72.2) | 62.9 (53.3-72.5) | 66.9 (60.3-73.5) | .83 |
| Other (including mainly Asians) | 65.4 (47.4-83.4) | 76.0 (59.8-92.2) | 75.0 (62.1-88.0) | 79.1 (71.3-86.9) | 74.8 (64.5-85.1) | 78.8 (69.3-88.4) | 77.6 (71.0-84.2) | .04 |
| **Education level** |  |  |  |  |  |  |  |  |
| <High school graduate | 51.2 (43.8-58.6) | 52.0 (43.6-60.4) | 55.9 (48.9-62.9) | 55.0 (44.6-65.5) | 60.0 (51.0-69.0) | 58.1 (47.0-69.2) | 57.2 (47.2-67.2) | .053 |
| High school graduate | 57.4 (51.8-63.0) | 59.1 (52.6-65.7) | 58.6 (51.5-65.8) | 59.0 (49.0-68.9) | 59.0 (49.8-68.1) | 56.2 (48.6-63.9) | 65.5 (56.8-74.2) | .06 |
| Some college or associate degree | 64.7 (59.4-69.9) | 67.7 (61.7-73.7) | 68.8 (62.8-74.8) | 65.3 (57.3-73.3) | 67.4 (61.1-73.6) | 69.7 (63.4-75.9) | 67.7 (61.1-74.2) | .09 |
| College graduate or above | 76.6 (70.4-82.8) | 78.6 (71.7-85.5) | 83.6 (78.3-88.9) | 84.1 (79.3-88.9) | 84.8 (80.1-89.5) | 85.1 (79.2-91.0) | 85.8 (80.4-91.2) | <.001 |
| **Marital status** |  |  |  |  |  |  |  |  |
| Married | 70.4 (64.4-76.5) | 70.3 (64.5-76.2) | 74.4 (69.6-79.2) | 75.7 (71.0-80.3) | 78.0 (73.5-82.5) | 75.4 (70.6-80.2) | 78.1 (73.9-82.3) | .004 |
| Divorced/separated/widowed | 57.6 (45.2-70.1) | 53.9 (44.6-63.2) | 60.1 (49.9-70.3) | 62.2 (53.1-71.3) | 57.5 (51.1-63.9) | 60.2 (47.8-72.7) | 63.9 (54.7-73.0) | .01 |
| Unmarried/cohabitation | 51.7 (43.6-59.7) | 60.4 (51.6-69.1) | 59.5 (50.1-68.8) | 62.1 (53.5-70.7) | 61.9 (55.2-68.6) | 64.1 (56.2-72.0) | 59.9 (47.1-72.7) | .07 |
| **Ratio of family income to poverty** |  |  |  |  |  |  |  |  |
| <1.30 | 53.5 (46.3-60.7) | 52.9 (45.0-60.8) | 55.5 (47.6-63.4) | 56.7 (46.5-66.9) | 55.0 (45.9-64.1) | 59.3 (51.5-67.1) | 56.8 (47.6-65.9) | .33 |
| 1.30-2.99 | 59.9 (54.1-65.6) | 64.8 (59.9-69.7) | 69.1 (63.0-75.3) | 66.6 (59.4-73.7) | 67.7 (61.0-74.3) | 67.8 (62.6-73.1) | 65.4 (57.0-73.8) | .37 |
| ≥3.00 | 69.9 (64.7-75.1) | 71.1 (64.9-77.2) | 74.0 (68.3-79.7) | 77.3 (72.0-82.5) | 77.5 (73.4-81.6) | 76.4 (70.1-82.7) | 80.6 (76.4-84.9) | <.001 |
| **Sleep health** |  |  |  |  |  |  |  |  |
| **Overall** | 83.7 (81.6-85.7) | 82.3 (79.8-84.9) | 83.4 (81.5-85.2) | 82.8 (80.3-85.3) | 83.3 (81.0-85.6) | 87.6 (85.4-89.8) | 84.1 (81.2-87.1) | .006 |
| **Sex** |  |  |  |  |  |  |  |  |
| Male | 83.3 (81.4-85.2) | 82.4 (79.4-85.4) | 82.8 (79.8-85.9) | 82.0 (78.8-85.2) | 83.2 (79.7-86.6) | 87.8 (85.2-90.3) | 83.0 (78.4-87.7) | .04 |
| Female | 84.1 (80.4-87.8) | 82.3 (79.1-85.4) | 83.8 (81.8-85.9) | 83.6 (80.4-86.7) | 83.5 (81.4-85.6) | 87.4 (84.8-90.0) | 85.1 (82.1-88.0) | .04 |
| **Age group, y** |  |  |  |  |  |  |  |  |
| 20-39 | 82.8 (81.0-84.7) | 80.8 (77.9-83.7) | 83.7 (81.9-85.6) | 83.1 (80.7-85.6) | 82.7 (80.0-85.5) | 86.2 (83.9-88.6) | 85.1 (82.0-88.2) | .02 |
| 40-64 | 83.3 (81.1-85.5) | 81.9 (79.6-84.3) | 82.7 (81.3-84.1) | 82.2 (79.7-84.6) | 81.5 (79.7-83.4) | 87.9 (86.4-89.4) | 82.9 (80.2-85.6) | .03 |
| 65-79 | 86.5 (84.3-88.7) | 87.0 (84.8-89.2) | 84.4 (81.4-87.3) | 83.6 (80.7-86.5) | 89.4 (87.0-91.8) | 90.0 (86.2-93.8) | 85.0 (81.7-88.3) | .90 |
| **Race/ethnicity** |  |  |  |  |  |  |  |  |
| Hispanic | 82.6 (77.9-87.3) | 83.4 (80.6-86.3) | 79.6 (76.3-82.9) | 82.1 (78.0-86.2) | 79.1 (75.0-83.2) | 84.5 (82.0-87.0) | 82.5 (78.0-87.0) | .33 |
| Non-Hispanic White | 85.3 (82.9-87.7) | 83.6 (80.2-87.0) | 86.1 (84.1-88.0) | 83.8 (80.9-86.8) | 85.5 (82.9-88.0) | 89.6 (86.6-92.5) | 85.3 (81.1-89.5) | .003 |
| Non-Hispanic Black | 72.1 (67.9-76.2) | 72.2 (66.9-77.6) | 70.0 (64.8-75.1) | 76.2 (72.5-80.0) | 75.3 (71.7-78.8) | 78.4 (73.8-83.0) | 77.3 (73.7-81.0) | <.001 |
| Other (including mainly Asians) | 79.4 (69.0-89.9) | 78.7 (68.9-88.6) | 84.4 (76.6-92.1) | 83.2 (79.4-87.0) | 81.4 (76.0-86.7) | 88.1 (82.9-93.4) | 86.1 (81.8-90.3) | .001 |
| **Education level** |  |  |  |  |  |  |  |  |
| <High school graduate | 78.5 (73.5-83.4) | 78.9 (75.1-82.8) | 79.7 (75.4-83.8) | 78.9 (74.8-83.0) | 79.0 (73.8-84.1) | 83.3 (77.2-89.4) | 78.0 (71.9-84.2) | .38 |
| High school graduate | 81.4 (77.9-85.0) | 80.6 (75.8-85.4) | 79.8 (75.7-83.8) | 79.1 (74.0-84.2) | 77.7 (73.8-81.5) | 82.5 (77.8-87.2) | 80.2 (74.0-86.4) | .97 |
| Some college or associate degree | 82.9 (79.5-86.4) | 80.0 (75.3-84.7) | 82.6 (79.3-85.9) | 81.5 (77.3-85.8) | 82.1 (78.3-85.9) | 87.4 (84.3-90.4) | 82.4 (78.2-86.7) | .02 |
| College graduate or above | 89.0 (85.6-92.4) | 88.7 (85.4-92.1) | 88.8 (86.3-91.2) | 87.7 (84.1-91.2) | 89.8 (86.6-93.1) | 92.4 (89.8-94.9) | 90.5 (87.1-93.9) | .003 |
| **Marital status** |  |  |  |  |  |  |  |  |
| Married | 85.8 (83.7-87.9) | 84.1 (81.3-86.8) | 85.3 (82.8-87.7) | 85.3 (81.9-88.7) | 85.4 (82.4-88.5) | 90.0 (87.7-92.3) | 86.1 (82.7-89.4) | .02 |
| Divorced/separated/widowed | 78.6 (73.3-84.0) | 75.8 (68.9-82.6) | 79.6 (75.2-84.0) | 77.8 (71.6-84.1) | 77.4 (71.4-83.4) | 83.2 (76.3-90.0) | 83.1 (78.4-87.8) | .22 |
| Unmarried/cohabitation | 79.9 (75.2-84.6) | 80.6 (76.0-85.1) | 80.9 (76.0-85.8) | 80.3 (75.8-84.9) | 81.7 (76.9-86.4) | 87.2 (83.8-90.6) | 81.2 (74.5-87.9) | .12 |
| **Ratio of family income to poverty** |  |  |  |  |  |  |  |  |
| <1.30 | 77.7 (72.2-83.2) | 77.9 (73.5-82.4) | 77.0 (72.0-82.1) | 78.7 (74.7-82.7) | 76.7 (72.7-80.6) | 81.1 (76.8-85.4) | 77.6 (71.2-84.1) | .46 |
| 1.30-2.99 | 80.8 (76.1-85.5) | 81.0 (77.5-84.4) | 82.0 (78.4-85.7) | 79.4 (74.9-83.8) | 81.1 (77.0-85.2) | 85.4 (81.4-89.4) | 83.1 (78.8-87.4) | .02 |
| ≥3.00 | 86.7 (84.1-89.3) | 84.8 (81.9-87.7) | 86.4 (83.9-89.0) | 86.2 (82.9-89.6) | 86.8 (83.9-89.7) | 91.0 (88.3-93.8) | 86.7 (82.9-90.4) | .02 |
| **Body mass index** |  |  |  |  |  |  |  |  |
| **Overall** | 63.4 (59.7-67.1) | 62.6 (59.3-66.0) | 60.7 (57.7-63.6) | 62.7 (58.8-66.6) | 60.6 (57.6-63.7) | 58.7 (55.0-62.4) | 56.2 (52.5-59.9) | <.001 |
| **Sex** |  |  |  |  |  |  |  |  |
| Male | 62.8 (59.0-66.7) | 62.0 (58.7-65.4) | 59.5 (55.0-64.0) | 62.1 (57.2-66.9) | 61.6 (57.5-65.8) | 59.7 (54.5-64.8) | 55.7 (50.4-61.1) | .001 |
| Female | 63.9 (58.7-69.0) | 63.0 (58.1-68.0) | 61.9 (58.2-65.6) | 63.3 (58.5-68.0) | 59.5 (55.3-63.7) | 57.8 (53.5-62.1) | 56.6 (50.2-62.9) | <.001 |
| **Age group, y** |  |  |  |  |  |  |  |  |
| 20-39 | 68.2 (64.2-72.1) | 65.2 (62.0-68.4) | 65.5 (62.1-68.9) | 66.9 (63.2-70.6) | 63.7 (61.1-66.3) | 61.5 (57.6-65.4) | 59.3 (54.6-63.9) | <.001 |
| 40-64 | 58.8 (55.3-62.3) | 60.5 (57.5-63.5) | 59.1 (57.0-61.2) | 59.2 (55.5-62.9) | 58.3 (55.3-61.3) | 56.6 (53.2-60.0) | 53.6 (50.8-56.4) | .003 |
| 65-79 | 64.5 (60.7-68.3) | 62.6 (57.9-67.3) | 53.8 (49.6-57.9) | 62.5 (57.6-67.3) | 59.9 (55.7-64.0) | 58.0 (53.7-62.3) | 56.0 (52.3-59.7) | <.001 |
| **Race/ethnicity** |  |  |  |  |  |  |  |  |
| Hispanic | 62.8 (55.9-69.7) | 57.9 (53.7-62.1) | 55.5 (49.7-61.3) | 54.7 (48.9-60.5) | 55.5 (50.0-61.0) | 51.7 (47.1-56.2) | 51.1 (44.8-57.3) | <.001 |
| Non-Hispanic White | 64.9 (60.4-69.4) | 64.0 (59.6-68.4) | 62.5 (59.3-65.8) | 64.7 (59.8-69.6) | 61.7 (57.7-65.8) | 60.1 (56.1-64.2) | 57.3 (52.3-62.2) | <.001 |
| Non-Hispanic Black | 53.6 (47.2-60.0) | 53.9 (49.5-58.4) | 50.1 (43.5-56.7) | 51.8 (46.2-57.3) | 51.5 (46.1-56.8) | 51.9 (45.0-58.7) | 51.3 (45.1-57.5) | .01 |
| Other (including mainly Asians) | 70.8 (58.7-82.9) | 76.9 (63.1-90.6) | 72.3 (61.6-82.9) | 76.9 (68.4-85.4) | 74.4 (67.3-81.6) | 68.7 (59.4-78.0) | 64.6 (58.0-71.3) | .16 |
| **Education Level** |  |  |  |  |  |  |  |  |
| <High school graduate | 63.2 (57.1-69.2) | 59.4 (54.7-64.0) | 56.8 (51.7-62.0) | 56.4 (49.0-63.9) | 56.3 (51.1-61.5) | 56.1 (47.8-64.5) | 56.3 (47.2-65.4) | .004 |
| High school graduate | 58.9 (53.2-64.5) | 61.2 (55.8-66.7) | 58.3 (53.0-63.5) | 58.4 (49.9-66.9) | 58.6 (52.5-64.7) | 55.2 (48.9-61.5) | 54.1 (47.5-60.7) | .004 |
| Some college or associate degree | 61.4 (56.5-66.4) | 60.5 (54.0-67.0) | 57.1 (53.2-60.9) | 61.5 (54.8-68.3) | 57.1 (52.2-62.0) | 55.0 (49.7-60.3) | 52.0 (47.9-56.1) | <.001 |
| College graduate or above | 69.8 (63.5-76.1) | 68.6 (63.9-73.4) | 68.1 (62.6-73.7) | 68.4 (62.6-74.2) | 66.7 (61.4-72.0) | 65.6 (60.5-70.7) | 62.1 (55.7-68.5) | .005 |
| **Marital status** |  |  |  |  |  |  |  |  |
| Married | 62.5 (57.6-67.5) | 61.5 (57.6-65.4) | 60.4 (57.3-63.4) | 63.0 (57.8-68.1) | 61.0 (56.8-65.1) | 57.5 (52.9-62.2) | 54.9 (51.3-58.5) | <.001 |
| Divorced/separated/widowed | 60.8 (50.6-70.9) | 61.4 (54.3-68.5) | 56.0 (48.4-63.7) | 57.0 (50.4-63.7) | 54.9 (47.3-62.6) | 54.7 (47.0-62.4) | 55.2 (45.4-65.0) | .02 |
| Unmarried/cohabitation | 65.8 (58.2-73.5) | 65.4 (59.5-71.4) | 58.5 (51.0-66.1) | 62.9 (55.3-70.5) | 62.1 (55.1-69.1) | 57.6 (50.8-64.5) | 54.8 (45.8-63.8) | <.001 |
| **Ratio of family income to poverty** |  |  |  |  |  |  |  |  |
| <1.30 | 59.0 (50.9-67.1) | 59.7 (54.8-64.7) | 57.3 (52.3-62.4) | 59.7 (54.5-64.9) | 59.0 (55.4-62.7) | 54.0 (48.3-59.7) | 55.3 (49.1-61.5) | .03 |
| 1.30-2.99 | 61.4 (55.8-67.1) | 61.1 (56.0-66.2) | 59.4 (54.4-64.4) | 61.1 (55.9-66.3) | 53.1 (47.0-59.1) | 59.9 (54.5-65.3) | 55.6 (49.1-62.2) | .004 |
| ≥3.00 | 65.5 (61.1-69.8) | 64.8 (60.1-69.4) | 62.6 (58.3-66.9) | 65.1 (59.1-71.2) | 64.5 (59.9-69.1) | 59.1 (53.8-64.4) | 57.1 (51.9-62.2) | <.001 |
| **Blood lipids** |  |  |  |  |  |  |  |  |
| **Overall** | 61.6 (59.1-64.0) | 61.0 (58.5-63.4) | 63.3 (61.0-65.6) | 62.8 (59.4-66.3) | 67.0 (64.2-69.9) | 65.2 (62.4-67.9) | 67.0 (63.5-70.4) | <.001 |
| **Sex** |  |  |  |  |  |  |  |  |
| Male | 58.6 (54.9-62.4) | 57.2 (54.6-59.8) | 59.9 (56.4-63.5) | 60.8 (56.8-64.9) | 64.8 (60.4-69.3) | 62.2 (58.2-66.2) | 65.4 (61.3-69.5) | <.001 |
| Female | 64.7 (61.4-68.0) | 64.6 (60.5-68.6) | 66.8 (64.1-69.6) | 65.2 (61.1-69.2) | 69.6 (66.5-72.7) | 68.5 (65.3-71.7) | 68.8 (63.8-73.8) | .002 |
| **Age group, y** |  |  |  |  |  |  |  |  |
| 20-39 | 70.6 (68.8-72.4) | 69.9 (67.3-72.5) | 73.2 (71.5-74.9) | 73.5 (70.5-76.6) | 75.5 (72.1-78.8) | 76.3 (73.8-78.7) | 76.5 (73.8-79.2) | <.001 |
| 40-64 | 55.2 (52.7-57.7) | 54.3 (52.1-56.4) | 55.9 (53.4-58.4) | 55.1 (51.9-58.3) | 61.1 (59.4-62.9) | 56.3 (54.3-58.4) | 61.7 (57.5-65.8) | <.001 |
| 65-79 | 57.7 (53.8-61.6) | 58.2 (55.2-61.2) | 60.4 (57.3-63.5) | 58.7 (53.7-63.6) | 63.3 (58.7-67.8) | 63.0 (57.7-68.2) | 59.1 (55.7-62.5) | .25 |
| **Race/ethnicity** |  |  |  |  |  |  |  |  |
| Hispanic | 60.4 (53.7-67.1) | 58.0 (54.0-61.9) | 59.6 (55.6-63.6) | 60.3 (55.9-64.7) | 63.5 (57.5-69.6) | 63.2 (58.6-67.7) | 64.2 (58.3-70.2) | <.001 |
| Non-Hispanic White | 61.0 (57.6-64.3) | 60.6 (57.5-63.7) | 63.3 (60.7-65.9) | 62.0 (57.2-66.8) | 67.1 (63.2-71.1) | 65.4 (61.5-69.2) | 67.8 (62.8-72.8) | <.001 |
| Non-Hispanic Black | 68.1 (63.8-72.5) | 67.7 (62.0-73.5) | 66.0 (60.7-71.3) | 69.3 (65.4-73.3) | 71.8 (66.9-76.7) | 71.4 (65.0-77.7) | 70.8 (65.9-75.7) | .001 |
| Other (including mainly Asians) | 57.1 (47.3-66.8) | 60.0 (48.5-71.6) | 72.0 (62.1-81.9) | 67.2 (59.9-74.5) | 67.9 (61.6-74.2) | 60.4 (52.5-68.3) | 64.0 (57.6-70.3) | .11 |
| **Education level** |  |  |  |  |  |  |  |  |
| <High school graduate | 60.8 (55.9-65.8) | 58.6 (52.6-64.6) | 60.2 (55.4-65.0) | 60.8 (53.8-67.8) | 67.7 (61.8-73.7) | 64.6 (59.0-70.2) | 64.5 (57.0-72.0) | <.001 |
| High school graduate | 57.6 (52.9-62.3) | 57.9 (53.4-62.3) | 58.8 (54.1-63.5) | 60.5 (53.7-67.3) | 66.8 (60.5-73.0) | 62.1 (56.6-67.6) | 66.0 (60.0-72.0) | <.001 |
| Some college or associate degree | 63.1 (57.4-68.7) | 61.5 (55.7-67.4) | 63.8 (60.2-67.4) | 63.1 (57.4-68.7) | 66.6 (62.3-70.9) | 65.0 (60.9-69.1) | 66.4 (60.6-72.1) | .01 |
| College graduate or above | 63.3 (57.1-69.5) | 63.8 (59.9-67.8) | 67.9 (63.7-72.0) | 64.5 (58.6-70.3) | 67.3 (62.3-72.4) | 67.3 (61.4-73.2) | 69.0 (62.7-75.3) | .03 |
| **Marital status** |  |  |  |  |  |  |  |  |
| Married | 60.3 (56.9-63.7) | 60.0 (56.9-63.0) | 62.4 (59.4-65.3) | 62.0 (58.0-65.9) | 65.1 (61.7-68.6) | 63.9 (60.3-67.4) | 66.4 (61.7-71.0) | <.001 |
| Divorced/separated/widowed | 60.9 (51.9-69.9) | 57.2 (51.9-62.5) | 60.5 (54.3-66.6) | 58.8 (52.9-64.6) | 66.3 (60.3-72.2) | 58.6 (51.0-66.1) | 67.6 (60.2-75.0) | .002 |
| Unmarried/cohabitation | 65.4 (59.3-71.5) | 64.2 (58.3-70.2) | 63.0 (57.2-68.9) | 63.8 (56.8-70.7) | 70.5 (64.7-76.3) | 67.5 (58.7-76.2) | 65.7 (58.0-73.4) | .005 |
| **Ratio of family income to poverty** |  |  |  |  |  |  |  |  |
| <1.30 | 61.1 (55.9-66.3) | 60.5 (55.2-65.8) | 61.3 (56.3-66.3) | 63.0 (56.7-69.3) | 67.0 (62.5-71.6) | 63.3 (57.8-68.9) | 67.5 (62.6-72.3) | <.001 |
| 1.30-2.99 | 60.8 (56.8-64.8) | 58.8 (54.5-63.2) | 62.3 (58.0-66.5) | 63.1 (58.4-67.8) | 66.6 (61.6-71.6) | 64.1 (59.9-68.4) | 68.2 (62.7-73.7) | <.001 |
| ≥3.00 | 61.8 (58.1-65.5) | 62.5 (58.0-67.1) | 64.5 (60.4-68.7) | 62.2 (57.3-67.1) | 67.0 (62.3-71.6) | 65.7 (61.6-69.9) | 66.6 (61.8-71.3) | .005 |
| **Blood glucose** |  |  |  |  |  |  |  |  |
| **Overall** | 83.9 (82.4-85.4) | 79.7 (77.3-82.0) | 80.1 (78.4-81.8) | 79.6 (77.7-81.5) | 81.1 (79.2-83.0) | 78.4 (75.6-81.1) | 77.4 (74.5-80.3) | <.001 |
| **Sex** |  |  |  |  |  |  |  |  |
| Male | 81.8 (79.6-84.0) | 77.2 (74.2-80.2) | 78.7 (76.2-81.2) | 77.1 (74.1-80.1) | 79.7 (76.8-82.6) | 76.0 (71.9-80.1) | 75.5 (71.7-79.3) | .002 |
| Female | 85.6 (83.0-88.3) | 81.9 (78.6-85.2) | 81.4 (79.1-83.7) | 82.1 (79.4-84.7) | 82.4 (80.3-84.5) | 80.5 (77.8-83.3) | 79.1 (75.0-83.3) | <.001 |
| **Age group, y** |  |  |  |  |  |  |  |  |
| 20-39 | 91.0 (90.2-91.8) | 87.9 (85.8-90.0) | 90.2 (89.1-91.3) | 88.9 (87.1-90.7) | 89.6 (88.2-91.0) | 89.1 (87.1-91.1) | 87.2 (85.0-89.4) | .03 |
| 40-64 | 81.1 (79.4-82.7) | 76.5 (74.7-78.3) | 77.0 (75.2-78.9) | 75.8 (74.0-77.7) | 78.1 (76.2-79.9) | 73.4 (70.3-76.4) | 73.6 (70.5-76.8) | <.001 |
| 65-79 | 74.9 (72.2-77.7) | 69.1 (64.7-73.4) | 65.0 (62.2-67.7) | 68.3 (66.0-70.5) | 69.7 (66.5-72.9) | 67.0 (63.3-70.7) | 64.8 (61.1-68.5) | <.001 |
| **Race/ethnicity** |  |  |  |  |  |  |  |  |
| Hispanic | 74.9 (68.9-80.8) | 76.5 (72.9-80.1) | 74.9 (71.3-78.5) | 73.6 (69.4-77.7) | 75.0 (70.9-79.1) | 72.3 (68.2-76.3) | 73.3 (69.6-77.0) | <.001 |
| Non-Hispanic White | 86.4 (84.6-88.3) | 80.8 (77.8-83.9) | 82.2 (79.9-84.6) | 82.1 (79.8-84.4) | 83.9 (81.5-86.2) | 80.9 (77.8-84.0) | 79.7 (76.2-83.3) | <.001 |
| Non-Hispanic Black | 76.4 (71.3-81.6) | 73.7 (69.7-77.8) | 73.8 (70.2-77.4) | 72.6 (69.3-75.9) | 75.2 (70.6-79.7) | 72.3 (68.4-76.2) | 72.0 (67.5-76.5) | .002 |
| Other (including mainly Asians) | 81.5 (70.8-92.2) | 77.6 (68.7-86.5) | 75.0 (67.9-82.1) | 76.4 (71.7-81.0) | 75.6 (71.2-80.0) | 74.1 (68.0-80.3) | 72.3 (66.7-77.8) | .02 |
| **Education level** |  |  |  |  |  |  |  |  |
| <High school graduate | 78.6 (73.6-83.6) | 75.3 (71.0-79.6) | 73.7 (70.3-77.2) | 72.8 (67.0-78.6) | 74.3 (70.0-78.7) | 69.9 (65.1-74.7) | 71.8 (65.7-77.9) | .002 |
| High school graduate | 82.7 (78.7-86.6) | 79.0 (75.0-83.0) | 78.8 (74.5-83.1) | 77.0 (72.3-81.6) | 79.9 (76.4-83.4) | 77.2 (72.6-81.9) | 78.3 (73.7-82.8) | .18 |
| Some college or associate degree | 83.1 (79.9-86.3) | 79.2 (75.3-83.0) | 80.2 (77.5-82.9) | 79.9 (76.8-83.1) | 80.1 (76.8-83.4) | 78.9 (74.3-83.6) | 75.9 (72.3-79.5) | .004 |
| College graduate or above | 88.3 (85.4-91.2) | 83.9 (79.7-88.1) | 84.2 (81.7-86.6) | 83.3 (79.8-86.8) | 85.3 (81.5-89.0) | 81.3 (77.3-85.3) | 79.9 (74.1-85.7) | .001 |
| **Marital status** |  |  |  |  |  |  |  |  |
| Married | 83.2 (80.8-85.5) | 80.0 (77.0-83.0) | 79.8 (77.7-82.0) | 78.8 (76.1-81.6) | 80.8 (77.8-83.9) | 78.3 (74.7-82.0) | 78.0 (74.3-81.7) | .001 |
| Divorced/separated/widowed | 83.7 (78.8-88.5) | 78.9 (74.1-83.8) | 80.4 (76.8-83.9) | 78.8 (73.9-83.7) | 81.3 (76.5-86.1) | 76.4 (69.9-82.9) | 75.8 (69.8-81.9) | .001 |
| Unmarried/cohabitation | 85.8 (82.5-89.2) | 78.8 (74.0-83.5) | 79.9 (76.8-82.9) | 80.4 (75.8-84.9) | 80.7 (74.4-87.0) | 77.4 (73.1-81.8) | 75.1 (68.1-82.2) | .009 |
| **Ratio of family income to poverty** |  |  |  |  |  |  |  |  |
| <1.30 | 79.9 (75.9-84.0) | 77.1 (72.6-81.6) | 76.7 (74.2-79.3) | 75.3 (71.6-78.9) | 77.1 (73.2-81.1) | 74.1 (70.2-77.9) | 73.9 (69.8-78.0) | .002 |
| 1.30-2.99 | 82.2 (78.2-86.3) | 79.0 (75.5-82.4) | 77.6 (74.6-80.7) | 78.3 (74.3-82.2) | 78.3 (74.8-81.8) | 75.7 (70.5-80.9) | 76.6 (72.0-81.1) | .002 |
| ≥3.00 | 85.5 (83.1-87.9) | 80.9 (77.7-84.0) | 82.6 (80.1-85.0) | 82.1 (79.5-84.7) | 83.7 (80.8-86.6) | 81.1 (77.2-85.1) | 78.6 (74.8-82.3) | <.001 |
| **Blood pressure** |  |  |  |  |  |  |  |  |
| **Overall** | 68.4 (65.2-71.5) | 71.3 (68.9-73.6) | 72.7 (69.9-75.5) | 70.4 (68.1-72.7) | 71.2 (69.0-73.4) | 70.2 (67.2-73.2) | 68.6 (65.3-71.9) | .35 |
| **Sex** |  |  |  |  |  |  |  |  |
| Male | 65.9 (61.3-70.5) | 66.9 (63.6-70.3) | 68.3 (64.4-72.2) | 66.6 (63.0-70.1) | 67.6 (63.7-71.4) | 66.9 (62.1-71.7) | 65.4 (60.7-70.1) | .56 |
| Female | 71.6 (68.3-74.8) | 75.1 (71.9-78.4) | 77.1 (74.2-80.1) | 74.4 (70.9-77.8) | 74.9 (71.7-78.0) | 73.9 (70.9-76.9) | 72.0 (68.2-75.8) | .26 |
| **Age group, y** |  |  |  |  |  |  |  |  |
| 20-39 | 83.6 (80.8-86.3) | 85.2 (83.8-86.7) | 84.7 (83.6-85.7) | 84.3 (82.0-86.6) | 85.3 (83.7-86.9) | 82.7 (79.9-85.4) | 82.2 (79.7-84.7) | .09 |
| 40-64 | 63.6 (60.4-66.8) | 67.2 (64.1-70.3) | 69.5 (66.0-72.9) | 64.7 (63.1-66.2) | 66.6 (64.3-68.9) | 66.7 (63.9-69.4) | 64.0 (60.1-67.8) | .78 |
| 65-79 | 46.3 (42.6-50.0) | 49.9 (47.4-52.3) | 54.0 (49.0-58.9) | 53.5 (49.3-57.8) | 50.7 (47.4-54.1) | 51.1 (46.7-55.4) | 49.6 (46.0-53.2) | .61 |
| **Race/ethnicity** |  |  |  |  |  |  |  |  |
| Hispanic | 71.8 (65.8-77.8) | 72.5 (68.4-76.6) | 71.2 (67.5-75.0) | 71.6 (66.1-77.0) | 73.7 (68.5-78.9) | 69.7 (66.2-73.2) | 66.5 (61.3-71.6) | <.001 |
| Non-Hispanic White | 69.0 (65.3-72.6) | 71.5 (68.3-74.6) | 74.0 (70.6-77.4) | 70.7 (67.7-73.6) | 72.6 (69.8-75.4) | 71.3 (67.2-75.4) | 70.8 (66.3-75.3) | .48 |
| Non-Hispanic Black | 61.0 (56.6-65.4) | 66.5 (61.1-71.9) | 61.6 (56.4-66.7) | 63.7 (59.5-67.9) | 61.9 (56.2-67.6) | 61.1 (54.9-67.4) | 57.6 (53.0-62.1) | <.001 |
| Other (including mainly Asians) | 65.6 (54.7-76.4) | 74.6 (66.1-83.0) | 76.3 (66.3-86.2) | 71.8 (64.8-78.9) | 67.6 (60.1-75.0) | 70.0 (63.5-76.4) | 65.3 (60.7-69.9) | .002 |
| **Education level** |  |  |  |  |  |  |  |  |
| <High school graduate | 67.0 (61.8-72.2) | 69.2 (65.2-73.2) | 71.3 (65.4-77.2) | 67.2 (62.0-72.3) | 68.2 (62.2-74.3) | 66.7 (60.1-73.4) | 62.5 (53.4-71.5) | .04 |
| High school graduate | 66.1 (61.2-71.0) | 68.3 (63.4-73.1) | 70.0 (66.0-74.1) | 68.6 (63.9-73.3) | 67.9 (63.6-72.3) | 65.8 (59.3-72.3) | 67.6 (61.6-73.6) | .95 |
| Some college or associate degree | 67.7 (61.4-74.1) | 70.5 (66.5-74.6) | 70.7 (66.5-74.8) | 70.8 (66.7-74.9) | 70.5 (65.7-75.2) | 71.2 (65.9-76.6) | 63.8 (59.0-68.6) | .29 |
| College graduate or above | 71.6 (66.5-76.6) | 76.7 (71.6-81.8) | 77.6 (72.3-83.0) | 72.5 (67.7-77.3) | 74.9 (70.7-79.0) | 73.1 (69.0-77.2) | 75.2 (69.5-81.0) | .98 |
| **Marital status** |  |  |  |  |  |  |  |  |
| Married | 68.1 (64.2-72.0) | 70.5 (67.0-74.1) | 72.5 (69.0-76.0) | 70.7 (67.6-73.7) | 71.3 (68.0-74.5) | 71.1 (66.6-75.5) | 69.4 (65.0-73.7) | .93 |
| Divorced/separated/widowed | 65.2 (58.5-72.0) | 71.0 (65.9-76.1) | 72.3 (66.6-78.0) | 69.5 (64.1-74.9) | 69.7 (63.9-75.6) | 66.9 (58.4-75.4) | 68.9 (60.8-77.1) | .37 |
| Unmarried/cohabitation | 73.5 (67.0-80.0) | 70.0 (64.9-75.2) | 74.0 (68.8-79.2) | 68.5 (62.4-74.6) | 72.9 (67.1-78.7) | 67.3 (60.6-74.0) | 61.1 (53.7-68.6) | <.001 |
| **Ratio of family income to poverty** |  |  |  |  |  |  |  |  |
| <1.30 | 66.8 (61.0-72.7) | 68.7 (64.8-72.6) | 69.7 (65.7-73.7) | 67.5 (62.5-72.5) | 69.0 (64.7-73.3) | 68.1 (62.4-73.7) | 65.2 (59.1-71.2) | .28 |
| 1.30-2.99 | 65.9 (58.9-72.9) | 71.3 (67.2-75.5) | 71.1 (67.6-74.6) | 69.3 (63.8-74.7) | 68.5 (65.2-71.8) | 69.4 (65.0-73.8) | 65.8 (61.3-70.3) | .44 |
| ≥3.00 | 69.8 (66.2-73.5) | 72.1 (68.2-76.1) | 74.5 (70.6-78.4) | 71.8 (68.3-75.4) | 72.8 (69.4-76.3) | 70.6 (66.2-75.1) | 70.6 (66.0-75.3) | .61 |

* Linear trends were estimated using linear regression model, with adjustment for sex, age, race/ethnicity, education level, marital status, and ratio of family income to poverty

*Note*: higher score denotes better cardiovascular health.

**Table S5.** Trends in crude mean scores (95% confidence interval) for individual CVH metrics for US adults, overall and by sex, age, race/ethnicity, education level, marital status and family income to poverty ratio category, 2005-2018

| **Characteristics** | 2005-2006 | 2007-2008 | 2009-2010 | 2011-2012 | 2013-2014 | 2015-2016 | 2017-2018 | *P* for trend ^*^ |
| --- | --- | --- | --- | --- | --- | --- | --- | --- |
| **Diet** |  |  |  |  |  |  |  |  |
| **Overall** | 40.4 (38.3-42.6) | 41.9 (37.9-45.8) | 44.8 (43.2-46.4) | 35.8 (33.6-37.9) | 44.5 (43.0-46.1) | 44.1 (40.6-47.7) | 41.3 (37.4-45.3) | .94 |
| **Sex** |  |  |  |  |  |  |  |  |
| Male | 36.2 (33.4-38.9) | 38.3 (34.7-41.9) | 40.5 (38.6-42.4) | 33.6 (31.8-35.4) | 40.4 (38.7-42.1) | 40.6 (36.3-45.0) | 38.1 (33.9-42.3) | .70 |
| Female | 44.4 (42.2-46.7) | 45.0 (40.0-49.9) | 48.8 (47.0-50.7) | 37.9 (34.7-41.1) | 48.5 (46.1-50.9) | 47.5 (43.1-52.0) | 44.4 (40.4-48.4) | .58 |
| **Age group, y** |  |  |  |  |  |  |  |  |
| 20-39 | 32.8 (29.8-35.9) | 34.6 (31.1-38.2) | 39.5 (36.5-42.5) | 32.1 (29.6-34.5) | 38.6 (35.4-41.7) | 38.5 (34.1-42.8) | 36.3 (31.4-41.2) | .23 |
| 40-64 | 43.4 (40.8-46.1) | 45.1 (40.1-50.1) | 46.6 (44.3-48.8) | 37.3 (34.7-40.0) | 46.8 (44.5-49.1) | 46.7 (43.1-50.3) | 42.8 (37.9-47.8) | .88 |
| 65-79 | 53.1 (49.2-56.9) | 54.7 (49.3-60.1) | 55.2 (52.9-57.5) | 42.2 (36.3-48.1) | 54.7 (50.3-59.1) | 51.0 (45.3-56.7) | 50.0 (44.4-55.7) | .02 |
| **Race/ethnicity** |  |  |  |  |  |  |  |  |
| Hispanic | 39.1 (35.1-43.0) | 42.9 (39.6-46.3) | 43.9 (39.7-48.1) | 37.1 (33.8-40.4) | 45.9 (41.5-50.3) | 41.4 (37.8-45.1) | 43.8 (38.8-48.8) | .86 |
| Non-Hispanic White | 41.6 (39.4-43.7) | 42.4 (37.0-47.8) | 45.5 (43.6-47.5) | 34.8 (32.1-37.5) | 43.9 (42.1-45.6) | 44.9 (40.8-49.1) | 40.5 (35.6-45.5) | .001 |
| Non-Hispanic Black | 33.5 (29.5-37.6) | 35.8 (32.6-39.0) | 38.7 (34.4-43.0) | 33.7 (29.0-38.4) | 39.0 (35.9-42.0) | 35.2 (31.0-39.3) | 37.8 (33.1-42.5) | .07 |
| Other (including mainly Asians) | 41.6 (35.6-47.6) | 43.5 (37.6-49.3) | 48.8 (44.1-53.6) | 44.8 (41.5-48.1) | 53.7 (48.2-59.2) | 51.8 (46.1-57.5) | 46.0 (41.6-50.3) | .11 |
| **Education Level** |  |  |  |  |  |  |  |  |
| <High school graduate | 36.2 (32.5-39.9) | 34.0 (30.0-38.0) | 40.0 (36.3-43.7) | 31.8 (28.2-35.4) | 39.1 (35.6-42.5) | 34.0 (29.2-38.8) | 36.9 (31.2-42.6) | .72 |
| High school graduate | 34.2 (30.2-38.1) | 36.0 (32.1-40.0) | 36.3 (33.7-38.8) | 31.0 (26.9-35.1) | 32.7 (29.7-35.7) | 36.9 (33.3-40.4) | 33.9 (29.1-38.6) | .69 |
| Some college or AA degree | 38.2 (35.1-41.3) | 42.0 (39.0-45.0) | 42.0 (39.9-44.1) | 32.8 (29.4-36.2) | 42.9 (39.1-46.6) | 41.9 (37.9-45.9) | 36.9 (31.8-41.9) | .61 |
| College graduate or above | 50.0 (46.2-53.8) | 51.8 (46.4-57.2) | 56.5 (53.7-59.4) | 42.7 (38.2-47.3) | 55.5 (52.4-58.6) | 54.1 (49.1-59.1) | 53.1 (49.0-57.1) | .50 |
| **Marital status** |  |  |  |  |  |  |  |  |
| Married | 41.3 (38.8-43.9) | 44.8 (41.2-48.4) | 47.5 (45.1-49.8) | 39.3 (36.4-42.3) | 47.2 (44.9-49.4) | 46.8 (43.3-50.3) | 43.7 (39.7-47.8) | .80 |
| Divorced/separated/widowed | 44.5 (41.2-47.8) | 41.4 (34.3-48.5) | 43.5 (38.4-48.7) | 32.3 (28.6-36.1) | 45.5 (42.0-49.1) | 44.0 (37.9-50.1) | 42.6 (39.2-45.9) | .69 |
| Unmarried/cohabitation | 35.5 (31.5-39.4) | 36.2 (31.8-40.6) | 39.9 (37.5-42.2) | 31.4 (28.1-34.6) | 38.3 (34.4-42.1) | 39.0 (34.2-43.9) | 36.3 (29.6-43.1) | .80 |
| **Ratio of family income to poverty** |  |  |  |  |  |  |  |  |
| <1.30 | 34.9 (31.1-38.7) | 35.8 (31.6-40.0) | 38.4 (35.8-41.0) | 30.2 (26.5-33.8) | 36.0 (32.7-39.3) | 35.8 (32.2-39.5) | 35.2 (31.6-38.9) | .55 |
| 1.30-2.99 | 36.2 (33.1-39.2) | 39.9 (35.2-44.5) | 41.6 (37.9-45.3) | 32.6 (29.3-35.9) | 40.3 (36.8-43.8) | 38.9 (36.2-41.6) | 38.5 (34.0-43.0) | .86 |
| ≥3.00 | 43.8 (41.0-46.7) | 45.2 (41.1-49.3) | 49.0 (47.0-51.0) | 40.0 (36.6-43.4) | 50.3 (48.1-52.5) | 50.3 (45.8-54.8) | 44.9 (40.1-49.7) | .79 |
| **Physical activity** |  |  |  |  |  |  |  |  |
| **Overall** | 57.9 (54.7-61.0) | 49.3 (43.1-55.5) | 49.3 (44.3-54.2) | 53.0 (47.9-58.0) | 50.9 (48.1-53.7) | 54.1 (50.3-57.9) | 53.2 (49.8-56.5) | .26 |
| **Sex** |  |  |  |  |  |  |  |  |
| Male | 58.5 (54.8-62.2) | 52.9 (47.0-58.9) | 54.0 (48.2-59.8) | 55.0 (49.3-60.7) | 52.0 (48.7-55.3) | 54.6 (49.9-59.2) | 54.9 (51.8-57.9) | .10 |
| Female | 57.3 (53.9-60.6) | 46.1 (39.0-53.3) | 44.8 (40.2-49.4) | 50.9 (45.1-56.7) | 49.8 (44.8-54.8) | 53.6 (49.0-58.2) | 51.6 (46.4-56.8) | .72 |
| **Age group, y** |  |  |  |  |  |  |  |  |
| 20-39 | 62.5 (58.9-66.2) | 57.6 (52.9-62.3) | 55.2 (48.5-61.9) | 60.8 (55.1-66.4) | 56.2 (50.7-61.6) | 59.5 (54.5-64.5) | 62.3 (57.8-66.7) | .79 |
| 40-64 | 56.3 (52.3-60.3) | 45.1 (36.9-53.3) | 47.2 (42.7-51.8) | 48.9 (43.3-54.5) | 48.7 (45.6-51.7) | 49.5 (44.5-54.5) | 47.3 (43.6-50.9) | .12 |
| 65-79 | 48.9 (41.3-56.5) | 36.7 (30.4-43.1) | 37.8 (33.2-42.4) | 42.7 (35.2-50.3) | 42.6 (35.8-49.4) | 54.2 (47.2-61.1) | 47.1 (41.4-52.7) | .97 |
| **Race/ethnicity** |  |  |  |  |  |  |  |  |
| Hispanic | 49.4 (41.6-57.1) | 38.8 (35.2-42.4) | 38.9 (34.1-43.8) | 41.8 (35.1-48.5) | 47.1 (41.9-52.4) | 44.6 (39.7-49.5) | 49.9 (43.5-56.2) | .45 |
| Non-Hispanic White | 60.1 (55.9-64.3) | 52.1 (42.6-61.7) | 52.5 (46.4-58.6) | 56.4 (50.3-62.5) | 51.4 (47.6-55.2) | 57.5 (53.7-61.2) | 54.8 (50.3-59.4) | .001 |
| Non-Hispanic Black | 50.9 (46.0-55.8) | 42.2 (37.0-47.4) | 40.4 (33.5-47.3) | 46.5 (40.8-52.1) | 46.7 (43.2-50.1) | 46.9 (40.8-52.9) | 47.4 (43.4-51.4) | .86 |
| Other (including mainly Asians) | 57.5 (45.7-69.2) | 51.1 (36.6-65.6) | 49.7 (42.2-57.3) | 52.7 (44.8-60.6) | 58.3 (52.0-64.5) | 52.7 (46.3-59.1) | 53.7 (46.2-61.3) | .14 |
| **Education Level** |  |  |  |  |  |  |  |  |
| <High school graduate | 38.0 (30.1-45.8) | 29.5 (24.6-34.3) | 26.1 (21.4-30.8) | 28.1 (21.4-34.8) | 30.4 (26.1-34.8) | 28.2 (22.1-34.3) | 29.4 (23.1-35.8) | .16 |
| High school graduate | 48.1 (43.9-52.3) | 42.6 (34.7-50.5) | 38.0 (33.1-42.8) | 46.1 (36.3-55.8) | 36.7 (33.8-39.6) | 43.4 (35.7-51.0) | 45.0 (41.1-49.0) | .64 |
| Some college or AA degree | 58.9 (54.7-63.1) | 52.0 (45.8-58.2) | 51.0 (45.0-56.9) | 51.1 (46.1-56.2) | 51.1 (46.9-55.3) | 53.9 (47.8-60.1) | 50.8 (46.8-54.8) | .42 |
| College graduate or above | 74.1 (70.3-77.8) | 65.0 (59.7-70.3) | 68.0 (60.6-75.4) | 68.1 (63.3-72.9) | 67.0 (63.3-70.7) | 69.3 (64.8-73.7) | 68.5 (63.7-73.3) | .84 |
| **Marital status** |  |  |  |  |  |  |  |  |
| Married | 57.1 (53.1-61.1) | 49.4 (41.7-57.1) | 47.8 (41.4-54.3) | 53.4 (48.3-58.6) | 51.4 (48.2-54.7) | 55.7 (51.3-60.1) | 53.2 (48.5-58.0) | .71 |
| Divorced/separated/widowed | 55.5 (49.8-61.1) | 38.1 (30.6-45.5) | 47.3 (42.9-51.7) | 45.4 (38.9-51.9) | 44.5 (40.5-48.5) | 41.3 (35.8-46.8) | 48.5 (42.6-54.4) | .36 |
| Unmarried/cohabitation | 61.4 (56.8-66.0) | 55.8 (50.8-60.8) | 53.3 (47.1-59.5) | 56.0 (49.0-63.0) | 53.7 (48.8-58.6) | 57.6 (51.9-63.3) | 55.7 (50.0-61.4) | .14 |
| **Ratio of family income to poverty** |  |  |  |  |  |  |  |  |
| <1.30 | 44.3 (39.9-48.7) | 35.1 (29.4-40.8) | 34.5 (28.6-40.4) | 40.8 (30.5-51.0) | 39.9 (35.5-44.3) | 43.1 (38.1-48.2) | 39.9 (33.6-46.3) | .98 |
| 1.30-2.99 | 51.5 (47.2-55.8) | 46.0 (37.1-54.8) | 42.7 (37.7-47.6) | 52.8 (46.1-59.4) | 45.8 (40.3-51.3) | 47.2 (41.1-53.3) | 46.7 (41.0-52.3) | .14 |
| ≥3.00 | 64.3 (61.0-67.5) | 56.3 (50.0-62.7) | 58.5 (52.1-64.9) | 58.6 (52.8-64.4) | 58.1 (55.0-61.3) | 62.2 (58.8-65.6) | 61.1 (56.5-65.7) | .54 |
| **Nicotine exposure** |  |  |  |  |  |  |  |  |
| **Overall** | 64.4 (62.4-66.3) | 65.3 (61.9-68.7) | 68.5 (65.0-72.0) | 68.9 (65.9-71.9) | 70.1 (67.4-72.9) | 70.5 (68.0-73.1) | 71.8 (69.1-74.6) | <.001 |
| **Sex** |  |  |  |  |  |  |  |  |
| Male | 59.3 (56.4-62.3) | 59.5 (55.8-63.3) | 62.8 (58.2-67.3) | 63.4 (58.8-68.0) | 67.0 (63.3-70.6) | 64.7 (61.5-67.9) | 65.7 (62.2-69.3) | .003 |
| Female | 69.1 (67.4-70.7) | 70.3 (66.4-74.3) | 73.8 (70.7-77.0) | 74.4 (71.8-77.0) | 73.2 (69.8-76.6) | 76.2 (73.1-79.3) | 77.6 (74.6-80.5) | .002 |
| **Age group, y** |  |  |  |  |  |  |  |  |
| 20-39 | 59.5 (55.7-63.3) | 61.0 (55.8-66.2) | 63.4 (57.4-69.4) | 68.0 (63.1-72.9) | 66.0 (61.7-70.2) | 66.6 (63.2-70.1) | 69.4 (65.1-73.8) | .001 |
| 40-64 | 66.0 (62.7-69.3) | 66.2 (62.6-69.8) | 70.0 (66.9-73.1) | 67.8 (64.1-71.6) | 71.0 (67.7-74.3) | 70.7 (66.4-75.0) | 72.1 (67.9-76.2) | .005 |
| 65-79 | 73.3 (69.2-77.4) | 77.6 (74.3-80.9) | 79.3 (76.5-82.0) | 77.0 (72.8-81.2) | 79.8 (76.6-83.0) | 80.4 (75.9-84.8) | 77.3 (73.2-81.4) | .66 |
| **Race/ethnicity** |  |  |  |  |  |  |  |  |
| Hispanic | 74.3 (70.4-78.3) | 70.6 (66.1-75.2) | 72.6 (69.1-76.1) | 74.5 (70.2-78.8) | 79.1 (75.9-82.4) | 74.8 (71.8-77.9) | 76.2 (71.1-81.3) | .01 |
| Non-Hispanic White | 63.3 (60.1-66.6) | 64.1 (59.0-69.3) | 67.9 (63.8-72.1) | 67.1 (63.4-70.8) | 68.4 (64.3-72.4) | 69.7 (66.3-73.1) | 70.8 (67.6-73.9) | .008 |
| Non-Hispanic Black | 61.3 (53.9-68.6) | 62.6 (58.4-66.8) | 63.8 (58.0-69.7) | 66.4 (61.0-71.7) | 65.2 (61.1-69.3) | 61.7 (54.1-69.3) | 66.5 (61.5-71.6) | .83 |
| Other (including mainly Asians) | 65.3 (56.8-73.8) | 73.6 (60.6-86.6) | 74.0 (64.3-83.7) | 78.3 (73.2-83.3) | 73.9 (67.6-80.2) | 78.1 (71.8-84.4) | 76.7 (72.2-81.3) | .04 |
| **Education Level** |  |  |  |  |  |  |  |  |
| <High school graduate | 51.5 (46.3-56.8) | 51.6 (45.3-57.9) | 55.5 (49.9-61.0) | 55.2 (49.2-61.2) | 59.4 (54.1-64.7) | 57.1 (48.9-65.3) | 57.2 (50.2-64.2) | .05 |
| High school graduate | 57.7 (55.3-60.0) | 58.8 (53.4-64.1) | 58.4 (54.1-62.8) | 59.3 (52.2-66.4) | 58.0 (51.6-64.3) | 55.8 (51.3-60.3) | 65.2 (59.9-70.5) | .06 |
| Some college or AA degree | 63.4 (59.9-66.8) | 66.5 (63.0-70.0) | 67.5 (63.3-71.7) | 64.5 (59.6-69.3) | 67.0 (62.7-71.2) | 69.5 (65.8-73.2) | 67.3 (64.2-70.4) | .09 |
| College graduate or above | 76.9 (73.1-80.7) | 78.5 (73.4-83.5) | 83.8 (79.6-88.1) | 83.9 (80.5-87.2) | 84.8 (81.2-88.5) | 85.0 (81.4-88.6) | 85.6 (82.9-88.3) | <.001 |
| **Marital status** |  |  |  |  |  |  |  |  |
| Married | 70.5 (67.7-73.3) | 70.5 (66.5-74.5) | 74.9 (71.5-78.3) | 75.5 (72.9-78.1) | 77.7 (74.9-80.5) | 75.9 (73.3-78.4) | 78.3 (75.8-80.7) | .004 |
| Divorced/separated/widowed | 59.2 (53.3-65.1) | 56.9 (51.4-62.3) | 61.8 (56.2-67.3) | 62.9 (57.6-68.2) | 60.2 (55.8-64.6) | 64.7 (56.9-72.5) | 68.5 (64.2-72.9) | .01 |
| Unmarried/cohabitation | 53.0 (50.4-55.6) | 59.8 (53.7-65.8) | 58.7 (52.8-64.6) | 60.6 (55.0-66.3) | 60.2 (55.9-64.5) | 63.4 (59.0-67.8) | 62.1 (56.3-67.9) | .07 |
| **Ratio of family income to poverty** |  |  |  |  |  |  |  |  |
| <1.30 | 51.9 (47.3-56.4) | 52.1 (45.3-58.9) | 54.2 (49.4-59.1) | 55.1 (47.3-62.8) | 55.4 (49.4-61.5) | 58.4 (53.5-63.3) | 55.5 (48.4-62.6) | .33 |
| 1.30-2.99 | 60.0 (57.6-62.4) | 64.8 (62.6-66.9) | 68.6 (64.5-72.7) | 66.3 (61.6-70.9) | 67.5 (62.0-73.0) | 67.3 (62.4-72.1) | 65.5 (59.7-71.3) | .37 |
| ≥3.00 | 69.6 (66.5-72.7) | 70.5 (66.7-74.3) | 74.2 (69.5-78.9) | 76.6 (72.7-80.4) | 77.9 (75.9-79.8) | 76.9 (72.5-81.3) | 80.8 (78.6-82.9) | <.001 |
| **Sleep health** |  |  |  |  |  |  |  |  |
| **Overall** | 83.5 (82.0-85.0) | 82.0 (79.9-84.1) | 83.3 (82.2-84.4) | 82.7 (81.0-84.4) | 83.0 (81.4-84.6) | 87.5 (85.9-89.2) | 84.1 (82.1-86.1) | .006 |
| **Sex** |  |  |  |  |  |  |  |  |
| Male | 82.9 (81.6-84.2) | 82.0 (79.4-84.7) | 82.8 (81.0-84.6) | 82.0 (80.1-83.9) | 82.6 (80.0-85.2) | 87.7 (86.1-89.2) | 83.1 (80.2-86.0) | .04 |
| Female | 84.1 (81.5-86.7) | 82.0 (80.1-83.9) | 83.7 (82.4-85.0) | 83.4 (81.3-85.6) | 83.3 (81.9-84.7) | 87.4 (85.4-89.5) | 85.1 (83.2-86.9) | .04 |
| **Age group, y** |  |  |  |  |  |  |  |  |
| 20-39 | 82.8 (81.0-84.7) | 80.8 (77.9-83.7) | 83.7 (81.9-85.6) | 83.1 (80.7-85.6) | 82.7 (80.0-85.5) | 86.2 (83.9-88.6) | 85.1 (82.0-88.2) | .02 |
| 40-64 | 83.3 (81.1-85.5) | 81.9 (79.6-84.3) | 82.7 (81.3-84.1) | 82.2 (79.7-84.6) | 81.5 (79.7-83.4) | 87.9 (86.4-89.4) | 82.9 (80.2-85.6) | .03 |
| 65-79 | 86.5 (84.3-88.7) | 87.0 (84.8-89.2) | 84.4 (81.4-87.3) | 83.6 (80.7-86.5) | 89.4 (87.0-91.8) | 90.0 (86.2-93.8) | 85.0 (81.7-88.3) | .90 |
| **Race/ethnicity** |  |  |  |  |  |  |  |  |
| Hispanic | 83.4 (80.9-85.9) | 83.1 (81.0-85.1) | 80.0 (77.6-82.3) | 82.3 (79.7-84.9) | 80.0 (76.6-83.3) | 84.2 (82.5-86.0) | 82.5 (79.6-85.4) | .33 |
| Non-Hispanic White | 85.3 (83.5-87.1) | 83.5 (80.5-86.4) | 85.9 (84.7-87.2) | 83.7 (81.7-85.8) | 85.2 (83.8-86.6) | 89.6 (87.6-91.7) | 85.3 (82.3-88.2) | .003 |
| Non-Hispanic Black | 71.6 (68.4-74.7) | 72.0 (68.4-75.6) | 69.2 (65.5-72.8) | 76.2 (73.6-78.7) | 74.9 (72.8-77.1) | 77.8 (74.6-81.0) | 77.0 (74.3-79.7) | <.001 |
| Other (including mainly Asians) | 83.5 (76.8-90.2) | 77.9 (71.5-84.3) | 84.2 (79.5-88.8) | 83.7 (80.7-86.7) | 81.4 (78.3-84.6) | 87.9 (84.9-90.9) | 86.5 (84.4-88.6) | .001 |
| **Education Level** |  |  |  |  |  |  |  |  |
| <High school graduate | 78.5 (75.0-82.1) | 78.9 (77.2-80.5) | 79.6 (77.4-81.7) | 79.0 (76.7-81.3) | 78.7 (75.7-81.8) | 83.2 (78.4-88.0) | 78.2 (74.9-81.5) | .38 |
| High school graduate | 81.5 (79.3-83.7) | 80.5 (76.3-84.6) | 79.6 (76.7-82.5) | 78.4 (75.7-81.2) | 77.1 (74.3-79.8) | 82.4 (79.5-85.3) | 80.1 (76.2-84.0) | .97 |
| Some college or AA degree | 82.4 (79.7-85.1) | 79 (75.8-82.1) | 82.8 (80.9-84.6) | 81.6 (78.8-84.4) | 81.7 (78.5-84.9) | 87.4 (85.5-89.2) | 82.5 (79.4-85.6) | .02 |
| College graduate or above | 88.7 (86.5-91.0) | 88.6 (86.6-90.5) | 88.5 (86.9-90.2) | 87.7 (85.9-89.4) | 89.6 (88.0-91.1) | 92.3 (90.5-94.1) | 90.6 (88.7-92.4) | .003 |
| **Marital status** |  |  |  |  |  |  |  |  |
| Married | 85.7 (84.0-87.5) | 83.9 (81.8-86.0) | 85.1 (83.9-86.3) | 85.2 (83.2-87.3) | 85.2 (83.3-87.1) | 90.2 (88.7-91.7) | 86.0 (83.7-88.3) | .02 |
| Divorced/separated/widowed | 79.4 (75.9-83.0) | 77.8 (73.6-81.9) | 79.7 (77.1-82.4) | 78.5 (76.4-80.6) | 77.6 (74.9-80.2) | 82.2 (78.4-86.1) | 81.6 (78.6-84.6) | .22 |
| Unmarried/cohabitation | 81.0 (79.0-83.0) | 80.7 (78.3-83.1) | 81.4 (78.5-84.3) | 80.6 (78.1-83.0) | 81.7 (79.3-84.0) | 85.2 (82.7-87.8) | 82.1 (78.3-85.9) | .12 |
| **Ratio of family income to poverty** |  |  |  |  |  |  |  |  |
| <1.30 | 77.4 (74.1-80.8) | 77.8 (74.8-80.8) | 77.4 (74.3-80.5) | 79.2 (76.5-82.0) | 77.0 (74.4-79.6) | 81.0 (78.8-83.2) | 78.1 (73.9-82.2) | .46 |
| 1.30-2.99 | 80.8 (78.1-83.4) | 80.7 (78.2-83.1) | 82.0 (79.4-84.6) | 79.3 (76.2-82.3) | 81.2 (77.9-84.4) | 85.6 (83.0-88.2) | 83.3 (80.9-85.8) | .02 |
| ≥3.00 | 86.4 (84.7-88.0) | 84.3 (82.1-86.5) | 86.3 (84.9-87.7) | 86.2 (83.5-88.8) | 86.5 (85.2-87.8) | 91.1 (89.3-92.9) | 86.6 (84.2-89.1) | .02 |
| **Body mass index** |  |  |  |  |  |  |  |  |
| **Overall** | 63.2 (60.1-66.3) | 62.6 (60.5-64.8) | 61.0 (59.0-62.9) | 62.6 (60.2-65.0) | 60.6 (58.9-62.3) | 58.7 (56.0-61.4) | 56.2 (53.8-58.7) | <.001 |
| **Sex** |  |  |  |  |  |  |  |  |
| Male | 63.0 (60.0-66.0) | 62.3 (60.0-64.7) | 59.6 (55.9-63.4) | 62.0 (59.3-64.8) | 61.9 (59.8-64.0) | 59.8 (56.0-63.7) | 55.9 (52.8-59.1) | .001 |
| Female | 63.4 (59.3-67.5) | 62.9 (59.7-66.0) | 62.2 (60.6-63.8) | 63.2 (60.4-66.0) | 59.3 (56.4-62.2) | 57.6 (54.8-60.4) | 56.5 (51.6-61.3) | <.001 |
| **Age group, y** |  |  |  |  |  |  |  |  |
| 20-39 | 68.2 (64.2-72.1) | 65.2 (62.0-68.4) | 65.5 (62.1-68.9) | 66.9 (63.2-70.6) | 63.7 (61.1-66.3) | 61.5 (57.6-65.4) | 59.3 (54.6-63.9) | <.001 |
| 40-64 | 58.8 (55.3-62.3) | 60.5 (57.5-63.5) | 59.1 (57.0-61.2) | 59.2 (55.5-62.9) | 58.3 (55.3-61.3) | 56.6 (53.2-60.0) | 53.6 (50.8-56.4) | .003 |
| 65-79 | 64.5 (60.7-68.3) | 62.6 (57.9-67.3) | 53.8 (49.6-57.9) | 62.5 (57.6-67.3) | 59.9 (55.7-64.0) | 58.0 (53.7-62.3) | 56.0 (52.3-59.7) | <.001 |
| **Race/ethnicity** |  |  |  |  |  |  |  |  |
| Hispanic | 62.5 (57.3-67.7) | 58.3 (55.3-61.2) | 56.3 (52.0-60.6) | 55.9 (52.8-59.1) | 55.5 (50.9-60.2) | 51.7 (48.4-55.0) | 51.0 (46.5-55.5) | <.001 |
| Non-Hispanic White | 64.4 (60.6-68.1) | 63.8 (60.9-66.7) | 62.4 (60.2-64.6) | 64.1 (60.8-67.5) | 61.5 (59.1-63.9) | 59.9 (57.4-62.4) | 57.0 (53.8-60.3) | <.001 |
| Non-Hispanic Black | 53.9 (49.5-58.3) | 54.2 (51.5-57.0) | 50.6 (44.9-56.3) | 51.8 (48.6-54.9) | 51.3 (48.8-53.9) | 52.1 (47.7-56.5) | 51.6 (47.6-55.5) | .01 |
| Other (including mainly Asians) | 68.0 (59.9-76.1) | 74.9 (65.8-84.0) | 72.6 (66.8-78.4) | 77.4 (73.5-81.4) | 74.1 (69.5-78.7) | 68.4 (60.3-76.5) | 63.2 (58.4-68.0) | .16 |
| **Education Level** |  |  |  |  |  |  |  |  |
| <High school graduate | 63.2 (60.0-66.4) | 59.5 (56.7-62.4) | 56.9 (53.2-60.6) | 56.6 (53.7-59.4) | 56.1 (53.7-58.4) | 55.9 (51.3-60.4) | 56.3 (51.2-61.3) | .004 |
| High school graduate | 58.7 (55.1-62.3) | 61.0 (57.6-64.4) | 57.7 (54.3-61.2) | 58.0 (52.0-64.1) | 58.6 (54.1-63.2) | 55.5 (51.4-59.5) | 54.1 (50.5-57.7) | .004 |
| Some college or AA degree | 61.5 (57.2-65.7) | 60.3 (56.5-64.1) | 58.0 (55.7-60.3) | 61.8 (57.1-66.5) | 57.4 (54.7-60.2) | 54.9 (50.7-59.2) | 51.8 (49.4-54.3) | <.001 |
| College graduate or above | 68.8 (63.5-74.0) | 68.3 (65.6-71.1) | 68.6 (64.3-72.9) | 68.3 (64.3-72.4) | 66.8 (63.3-70.3) | 65.3 (61.5-69.1) | 62.2 (58.1-66.2) | .005 |
| **Marital status** |  |  |  |  |  |  |  |  |
| Married | 61.9 (58.2-65.6) | 60.9 (58.7-63.2) | 60.5 (59.0-61.9) | 62.5 (59.2-65.7) | 60.5 (58.1-63.0) | 57.8 (54.5-61.2) | 54.7 (52.5-56.9) | <.001 |
| Divorced/separated/widowed | 59.1 (55.6-62.7) | 62.0 (57.1-67.0) | 55.1 (50.4-59.8) | 56.8 (52.3-61.2) | 55.1 (51.6-58.6) | 54.7 (50.2-59.2) | 55.7 (51.2-60.2) | .02 |
| Unmarried/cohabitation | 69.2 (65.1-73.2) | 66.4 (62.9-69.9) | 65.2 (60.7-69.7) | 65.8 (62.6-69.0) | 64.2 (60.9-67.5) | 62.5 (58.9-66.1) | 59.2 (54.3-64.1) | <.001 |
| **Ratio of family income to poverty** |  |  |  |  |  |  |  |  |
| <1.30 | 60.1 (53.9-66.4) | 60.2 (56.5-63.9) | 58.7 (55.3-62.0) | 60.9 (57.5-64.4) | 60.2 (58.4-61.9) | 55.1 (51.2-59.0) | 56.0 (51.4-60.7) | .03 |
| 1.30-2.99 | 61.9 (57.3-66.4) | 61.2 (59.1-63.4) | 59.8 (56.2-63.3) | 61.1 (58.5-63.7) | 53.7 (49.9-57.5) | 60.6 (57.1-64.1) | 55.4 (51.3-59.5) | .004 |
| ≥3.00 | 64.6 (61.6-67.7) | 64.3 (61.1-67.5) | 62.5 (59.6-65.4) | 64.2 (60.2-68.1) | 64.0 (60.9-67.2) | 58.9 (55.4-62.4) | 56.7 (53.7-59.7) | <.001 |
| **Blood lipids** |  |  |  |  |  |  |  |  |
| **Overall** | 61.6 (60.0-63.2) | 61.1 (59.4-62.7) | 63.1 (61.8-64.5) | 62.9 (60.3-65.5) | 67.0 (65.2-68.9) | 65.1 (63.9-66.2) | 67.2 (64.3-70.0) | <.001 |
| **Sex** |  |  |  |  |  |  |  |  |
| Male | 58.8 (56.7-60.8) | 57.0 (55.1-59.0) | 59.6 (57.9-61.3) | 60.8 (57.8-63.8) | 64.7 (61.8-67.6) | 62.3 (60.2-64.3) | 65.7 (62.4-69.0) | <.001 |
| Female | 64.3 (62.1-66.4) | 64.6 (61.4-67.7) | 66.5 (64.3-68.6) | 64.9 (61.7-68.0) | 69.3 (67.2-71.4) | 67.8 (66.0-69.5) | 68.5 (64.7-72.4) | .002 |
| **Age group, y** |  |  |  |  |  |  |  |  |
| 20-39 | 70.6 (68.8-72.4) | 69.9 (67.3-72.5) | 73.2 (71.5-74.9) | 73.5 (70.5-76.6) | 75.5 (72.1-78.8) | 76.3 (73.8-78.7) | 76.5 (73.8-79.2) | <.001 |
| 40-64 | 55.2 (52.7-57.7) | 54.3 (52.1-56.4) | 55.9 (53.4-58.4) | 55.1 (51.9-58.3) | 61.1 (59.4-62.9) | 56.3 (54.3-58.4) | 61.7 (57.5-65.8) | <.001 |
| 65-79 | 57.7 (53.8-61.6) | 58.2 (55.2-61.2) | 60.4 (57.3-63.5) | 58.7 (53.7-63.6) | 63.3 (58.7-67.8) | 63.0 (57.7-68.2) | 59.1 (55.7-62.5) | .25 |
| **Race/ethnicity** |  |  |  |  |  |  |  |  |
| Hispanic | 63.4 (59.3-67.5) | 60.9 (58.6-63.1) | 61.4 (59.2-63.5) | 62.9 (60.4-65.5) | 64.7 (60.9-68.5) | 64.8 (61.7-67.9) | 65.7 (62.0-69.4) | <.001 |
| Non-Hispanic White | 60.4 (58.1-62.7) | 60.2 (58.3-62.1) | 62.3 (60.6-64.1) | 61.0 (57.5-64.5) | 66.6 (64.0-69.2) | 64.5 (63.2-65.7) | 67.2 (62.9-71.4) | <.001 |
| Non-Hispanic Black | 69.1 (66.4-71.7) | 68.5 (64.0-73.0) | 66.9 (63.5-70.3) | 70.3 (68.2-72.3) | 72.0 (69.1-75.0) | 72.7 (68.9-76.5) | 72.2 (68.7-75.7) | .001 |
| Other (including mainly Asians) | 59.2 (52.8-65.5) | 59.0 (50.3-67.6) | 70.5 (64.0-77.0) | 68.6 (63.9-73.3) | 68.2 (63.6-72.9) | 62.0 (55.8-68.2) | 64.3 (60.9-67.8) | .11 |
| **Education Level** |  |  |  |  |  |  |  |  |
| <High school graduate | 61.3 (58.4-64.2) | 59.3 (56.7-61.9) | 59.6 (56.4-62.7) | 61.1 (57.6-64.6) | 67.5 (64.0-71.0) | 65.0 (61.7-68.2) | 64.4 (59.9-69.0) | <.001 |
| High school graduate | 57.3 (54.1-60.5) | 57.5 (54.9-60.0) | 58.2 (54.6-61.9) | 59.4 (53.9-64.8) | 66.4 (62.4-70.4) | 62.4 (58.3-66.5) | 66.1 (61.8-70.4) | <.001 |
| Some college or AA degree | 64.1 (61.2-67.0) | 62.7 (58.9-66.4) | 64.3 (61.9-66.7) | 63.8 (58.6-69.0) | 67.5 (64.7-70.3) | 65.1 (62.4-67.8) | 66.7 (62.7-70.8) | .01 |
| College graduate or above | 62.5 (57.6-67.4) | 63.6 (60.9-66.3) | 67.5 (65.7-69.3) | 64.6 (61.0-68.3) | 66.8 (63.6-69.9) | 66.7 (63.2-70.2) | 69.1 (64.5-73.8) | .03 |
| **Marital status** |  |  |  |  |  |  |  |  |
| Married | 59.6 (57.8-61.4) | 58.5 (57.0-60.0) | 60.8 (59.1-62.6) | 60.7 (58.2-63.3) | 64.0 (62.1-65.9) | 62.3 (60.3-64.2) | 65.0 (61.2-68.8) | <.001 |
| Divorced/separated/widowed | 56.9 (53.7-60.0) | 55.4 (52.5-58.3) | 58.4 (54.5-62.2) | 55.6 (52.1-59.1) | 63.7 (61.3-66.2) | 56.8 (52.2-61.5) | 63.7 (59.2-68.2) | .002 |
| Unmarried/cohabitation | 69.8 (66.7-72.9) | 69.6 (66.5-72.7) | 70.6 (67.6-73.5) | 70.2 (65.6-74.7) | 75.6 (71.7-79.4) | 74.7 (70.6-78.8) | 72.9 (69.2-76.6) | .005 |
| **Ratio of family income to poverty** |  |  |  |  |  |  |  |  |
| <1.30 | 63.5 (60.3-66.7) | 62.8 (58.9-66.7) | 63.3 (60.0-66.6) | 66.2 (61.6-70.7) | 69.7 (66.9-72.5) | 65.9 (62.9-68.9) | 69.4 (67.1-71.8) | <.001 |
| 1.30-2.99 | 61.7 (59.6-63.9) | 59.7 (57.0-62.4) | 62.9 (60.1-65.7) | 63.4 (60.4-66.4) | 67.4 (64.0-70.8) | 66.0 (63.7-68.3) | 68.5 (64.7-72.3) | <.001 |
| ≥3.00 | 61.0 (58.7-63.4) | 61.1 (58.8-63.5) | 63.2 (60.1-66.3) | 61.1 (58.4-63.8) | 65.7 (63.4-68.0) | 64.2 (62.5-65.9) | 65.7 (61.7-69.7) | .005 |
| **Blood glucose** |  |  |  |  |  |  |  |  |
| **Overall** | 84.2 (82.9-85.6) | 80.4 (78.8-81.9) | 80.8 (79.8-81.8) | 80.2 (78.6-81.8) | 81.6 (80.3-82.9) | 78.6 (76.6-80.5) | 77.7 (75.9-79.4) | <.001 |
| **Sex** |  |  |  |  |  |  |  |  |
| Male | 82.9 (81.5-84.2) | 78.2 (75.8-80.5) | 79.8 (78.6-81.0) | 78.2 (76.4-80.1) | 80.8 (78.8-82.8) | 77.0 (74.5-79.5) | 76.3 (73.2-79.5) | .002 |
| Female | 85.5 (83.9-87.2) | 82.3 (80.2-84.4) | 81.7 (80.0-83.4) | 82.2 (80.0-84.4) | 82.4 (81.2-83.6) | 80.1 (78.2-82.0) | 79.0 (76.6-81.3) | <.001 |
| **Age group, y** |  |  |  |  |  |  |  |  |
| 20-39 | 91.0 (90.2-91.8) | 87.9 (85.8-90.0) | 90.2 (89.1-91.3) | 88.9 (87.1-90.7) | 89.6 (88.2-91.0) | 89.1 (87.1-91.1) | 87.2 (85.0-89.4) | .03 |
| 40-64 | 81.1 (79.4-82.7) | 76.5 (74.7-78.3) | 77.0 (75.2-78.9) | 75.8 (74.0-77.7) | 78.1 (76.2-79.9) | 73.4 (70.3-76.4) | 73.6 (70.5-76.8) | <.001 |
| 65-79 | 74.9 (72.2-77.7) | 69.1 (64.7-73.4) | 65.0 (62.2-67.7) | 68.3 (66.0-70.5) | 69.7 (66.5-72.9) | 67.0 (63.3-70.7) | 64.8 (61.1-68.5) | <.001 |
| **Race/ethnicity** |  |  |  |  |  |  |  |  |
| Hispanic | 78.2 (74.6-81.9) | 80.9 (78.8-83.0) | 78.4 (75.8-81.0) | 77.4 (75.1-79.7) | 78.4 (75.3-81.6) | 75.6 (73.1-78.0) | 76.5 (75.0-78.0) | <.001 |
| Non-Hispanic White | 86.2 (84.7-87.8) | 81.0 (78.9-83.1) | 82.0 (80.6-83.5) | 82.0 (80.0-84.0) | 83.8 (82.3-85.3) | 80.0 (77.8-82.2) | 79.1 (77.1-81.1) | <.001 |
| Non-Hispanic Black | 78.0 (74.3-81.7) | 76.0 (72.8-79.2) | 75.5 (72.4-78.7) | 74.3 (71.8-76.8) | 76.1 (73.0-79.1) | 74.2 (71.1-77.3) | 73.9 (71.2-76.7) | .002 |
| Other (including mainly Asians) | 80.6 (73.5-87.8) | 78.7 (70.9-86.5) | 80.4 (76.0-84.7) | 78.3 (74.5-82.1) | 77.6 (74.4-80.8) | 77.1 (71.6-82.7) | 74.7 (70.7-78.6) | .02 |
| **Education Level** |  |  |  |  |  |  |  |  |
| <High school graduate | 78.7 (75.7-81.7) | 76.4 (73.4-79.4) | 73.6 (71.2-76.0) | 72.9 (70.3-75.6) | 74.8 (71.2-78.3) | 70.6 (69.1-72.2) | 71.6 (67.2-76.0) | .002 |
| High school graduate | 82.4 (79.5-85.2) | 79.0 (76.2-81.8) | 78.8 (75.9-81.8) | 76.7 (73.3-80.1) | 80.7 (78.5-82.8) | 77.8 (74.0-81.5) | 78.6 (75.6-81.5) | .18 |
| Some college or AA degree | 84.2 (82.4-85.9) | 80.5 (77.9-83.1) | 81.9 (80.2-83.6) | 81.4 (78.7-84.2) | 81.0 (79.4-82.6) | 79.1 (75.7-82.6) | 76.4 (74.3-78.6) | .004 |
| College graduate or above | 88.4 (86.1-90.7) | 83.9 (81.1-86.7) | 84.8 (83.1-86.5) | 83.9 (81.1-86.6) | 85.3 (82.5-88.1) | 81.0 (78.2-83.9) | 79.7 (75.6-83.8) | .001 |
| **Marital status** |  |  |  |  |  |  |  |  |
| Married | 82.8 (81.1-84.5) | 79.4 (77.3-81.6) | 79.0 (77.8-80.2) | 78.2 (76.4-80.1) | 80.0 (78.0-82.0) | 76.8 (74.3-79.2) | 76.7 (74.3-79.1) | .001 |
| Divorced/separated/widowed | 81.2 (77.5-84.8) | 76.1 (73.4-78.8) | 76.8 (74.2-79.4) | 76.9 (74.7-79.1) | 77.6 (74.8-80.4) | 71.8 (67.5-76.1) | 71.3 (66.6-76.0) | .001 |
| Unmarried/cohabitation | 89.8 (88.4-91.3) | 84.8 (81.6-88.0) | 86.7 (85.3-88.0) | 85.3 (82.8-87.9) | 87.4 (84.8-89.9) | 85.5 (83.1-87.8) | 83.0 (79.6-86.4) | .009 |
| **Ratio of family income to poverty** |  |  |  |  |  |  |  |  |
| <1.30 | 81.7 (79.1-84.3) | 80.2 (77.2-83.2) | 79.5 (77.7-81.2) | 78.5 (74.8-82.2) | 80.4 (78.7-82.1) | 76.9 (73.8-79.9) | 77.8 (75.5-80.1) | .002 |
| 1.30-2.99 | 82.8 (79.9-85.7) | 79.7 (77.7-81.7) | 78.7 (76.4-81.0) | 78.9 (76.9-80.9) | 79.3 (77.0-81.7) | 77.6 (73.8-81.5) | 77.2 (75.0-79.4) | .002 |
| ≥3.00 | 85.5 (83.8-87.2) | 80.8 (79.2-82.4) | 82.4 (80.7-84.0) | 81.7 (79.7-83.7) | 83.2 (80.9-85.5) | 79.7 (77.0-82.4) | 77.9 (75.2-80.6) | <.001 |
| **Blood pressure** |  |  |  |  |  |  |  |  |
| **Overall** | 69.3 (67.4-71.2) | 72.7 (70.9-74.5) | 73.6 (71.7-75.5) | 71.3 (69.0-73.5) | 72.0 (70.8-73.3) | 70.6 (68.8-72.4) | 69.0 (66.9-71.1) | .35 |
| **Sex** |  |  |  |  |  |  |  |  |
| Male | 67.1 (64.3-69.9) | 68.8 (66.6-71.0) | 69.1 (66.6-71.7) | 67.9 (65.1-70.6) | 69.0 (66.3-71.7) | 67.8 (64.7-70.9) | 66.1 (63.6-68.6) | .56 |
| Female | 71.4 (69.8-73.0) | 76.1 (73.9-78.4) | 77.8 (75.6-79.9) | 74.6 (72.2-77.1) | 75.0 (72.9-77.1) | 73.4 (71.8-75.0) | 71.7 (68.8-74.7) | .26 |
| **Age group, y** |  |  |  |  |  |  |  |  |
| 20-39 | 83.6 (80.8-86.3) | 85.2 (83.8-86.7) | 84.7 (83.6-85.7) | 84.3 (82.0-86.6) | 85.3 (83.7-86.9) | 82.7 (79.9-85.4) | 82.2 (79.7-84.7) | .08 |
| 40-64 | 63.6 (60.4-66.8) | 67.2 (64.1-70.3) | 69.5 (66.0-72.9) | 64.7 (63.1-66.2) | 66.6 (64.3-68.9) | 66.7 (63.9-69.4) | 64.0 (60.1-67.8) | .78 |
| 65-79 | 46.3 (42.6-50.0) | 49.9 (47.4-52.3) | 54.0 (49.0-58.9) | 53.5 (49.3-57.8) | 50.7 (47.4-54.1) | 51.1 (46.7-55.4) | 49.6 (46.0-53.2) | .61 |
| **Race/ethnicity** |  |  |  |  |  |  |  |  |
| Hispanic | 76.5 (73.3-79.7) | 77.6 (75.3-79.8) | 75.9 (73.1-78.7) | 77.7 (74.2-81.3) | 77.6 (74.2-80.9) | 74.1 (72.0-76.3) | 70.8 (67.6-73.9) | <.001 |
| Non-Hispanic White | 68.8 (66.5-71.0) | 71.9 (69.5-74.4) | 73.9 (71.6-76.2) | 70.3 (67.4-73.2) | 72.3 (70.3-74.2) | 70.4 (68.0-72.8) | 69.9 (67.0-72.7) | .48 |
| Non-Hispanic Black | 64.1 (61.7-66.6) | 69.8 (65.5-74.0) | 64.7 (60.2-69.1) | 65.8 (62.4-69.3) | 64.1 (60.6-67.5) | 64.0 (60.6-67.4) | 60.7 (57.3-64.2) | <.001 |
| Other (including mainly Asians) | 73.3 (64.2-82.4) | 77.3 (72.0-82.5) | 80.5 (76.1-84.9) | 75.4 (71.4-79.4) | 70.6 (65.5-75.6) | 72.9 (68.4-77.4) | 69.1 (66.4-71.7) | .002 |
| **Education Level** |  |  |  |  |  |  |  |  |
| <High school graduate | 67.4 (63.7-71.2) | 70.4 (67.6-73.3) | 71.3 (67.6-75.1) | 67.3 (62.8-71.8) | 69.2 (64.6-73.7) | 68.1 (63.5-72.6) | 62.2 (57.2-67.2) | .04 |
| High school graduate | 65.5 (62.6-68.4) | 68.6 (64.4-72.7) | 70.3 (67.4-73.1) | 68.1 (64.4-71.8) | 69.1 (65.8-72.5) | 66.4 (62.6-70.3) | 68.2 (63.9-72.6) | .95 |
| Some college or AA degree | 70.7 (67.0-74.3) | 72.7 (70.5-75.0) | 72.8 (70.5-75.0) | 72.7 (70.0-75.4) | 72.0 (70.0-74.1) | 71.6 (68.8-74.4) | 65.1 (61.3-68.9) | .29 |
| College graduate or above | 71.9 (68.3-75.4) | 77.7 (74.9-80.6) | 78.2 (74.7-81.7) | 73.3 (69.2-77.3) | 74.9 (71.8-78.0) | 73.0 (69.8-76.3) | 75.2 (70.7-79.7) | .98 |
| **Marital status** |  |  |  |  |  |  |  |  |
| Married | 67.7 (64.8-70.6) | 70.0 (67.3-72.7) | 71.4 (68.8-74.0) | 69.6 (67.2-72.0) | 69.8 (67.6-72.0) | 69.3 (66.3-72.3) | 67.6 (65.1-70.1) | .93 |
| Divorced/separated/widowed | 59.1 (55.1-63.1) | 66.6 (63.4-69.9) | 69.3 (65.1-73.5) | 64.3 (60.2-68.4) | 64.5 (60.8-68.1) | 62.3 (58.8-65.8) | 63.8 (59.7-68.0) | .37 |
| Unmarried/cohabitation | 80.5 (78.1-82.8) | 81.9 (80.0-83.8) | 80.6 (78.5-82.7) | 77.7 (74.4-81.1) | 81.6 (78.8-84.3) | 77.4 (75.2-79.6) | 74.4 (72.0-76.8) | <.001 |
| **Ratio of family income to poverty** |  |  |  |  |  |  |  |  |
| <1.30 | 70.7 (66.0-75.5) | 73.8 (70.4-77.2) | 73.7 (71.8-75.6) | 73.1 (67.8-78.5) | 74.5 (72.8-76.2) | 71.6 (68.6-74.7) | 70.5 (66.6-74.4) | .28 |
| 1.30-2.99 | 67.2 (63.1-71.3) | 72.5 (69.4-75.6) | 72.3 (70.3-74.3) | 70.1 (66.1-74.2) | 70.0 (68.2-71.7) | 72.0 (69.9-74.1) | 66.6 (64.1-69.0) | .44 |
| ≥3.00 | 69.9 (68.1-71.7) | 72.4 (69.7-75.2) | 74.2 (71.6-76.8) | 71.0 (67.9-74.2) | 71.9 (69.7-74.1) | 69.4 (66.6-72.3) | 69.6 (66.3-73.0) | .61 |

* Linear trends were examined using linear regression, with adjustment for sex, age, race/ethnicity, education level, marital status, and ratio of family income to poverty.

*Note*: higher score denotes better cardiovascular health
